# Prevalence and predictors of virological failure among the people living with HIV on antiretroviral treatment in East Africa: evidence from a systematic review with meta-analysis and meta-regression of published studies from 2016 to 2023

**DOI:** 10.1080/25787489.2025.2490774

**Published:** 2025-04-11

**Authors:** Maria Magdalene Namaganda, Hussein Mukasa Kafeero, Joyce Nakatumba Nabende, David Patrick Kateete, Charles Batte, Misaki Wanyengera, Daudi Jjingo, Moses Joloba, Florence Kivunike, Isaac Ssewanyana, Yunus Miya, Darius Kato, Simple Ouma, Frederick Elishama Kakembo, Stephen Kanyerezi, Jupiter Marina Kabahiita, Fahad Muwanda, Gerald Mboowa

**Affiliations:** a Department of Immunology and Molecular Biology, School of Biomedical Sciences, College of Health Sciences, Makerere University, Kampala, Uganda; b The African Center of Excellence in Bioinformatics and Data-Intensive Science (ACE), Kampala, Uganda; c Department of Medical Microbiology, Habib Medical School, Faculty of Health Sciences, Islamic University in Uganda, Kampala, Uganda; d Department of Computer Science, School of Computing and Information Technology, Makerere University, Kampala, Uganda; e Uganda National Health Laboratory Services, Kampala, Uganda; f The AIDS Support Organization (TASO), Kampala, Uganda

**Keywords:** Predictors, prevalence, HIV, virological failure, East Africa

## Abstract

**Background:**

Virological failure (VF) significantly threatens the efficacy of antiretroviral therapy (ART) programs in East Africa. This systematic review and meta-analysis assess the prevalence and predictors of VF among individuals living with HIV.

**Methods:**

We searched PubMed, Web of Science, African Journals Online, and EMBASE for relevant studies. Heterogeneity was assessed using the *I*^2^ statistic, and random-effects models addressed between-study variability. Publication bias was examined through funnel plots, Egger’s regression, and Begg’s tests. Subgroup analyses and meta-regression explored heterogeneity sources and potential VF predictors. Analyses were conducted using MedCalc version 20.010, adhering to PRISMA 2020 guidelines.

**Results:**

Twenty-five records were included, with a sample size of 29,829 people living with HIV on ART. The pooled prevalence of VF in East Africa was 19.4% (95% CI: 15.2%–24.0%), with substantial heterogeneity across studies. Sociodemographic predictors of VF included male sex (30.9%, *p* < .001), unmarried status (28.2%, *p* < .001), lower educational attainment (33.0%, *p* < .001), non-formal employment (47.2%, *p* < .001), and urban residence (51.2%, *p* < .001). Clinical factors associated with higher VF rates were ambulatory status (44.7%, *p* < .001), low CD4 count (35.1%, *p* < .001), low haemoglobin (52.2%, *p* < .001), advanced HIV stage III/IV (44.2%, *p* < .001), HIV/TB co-infection (24.3%, *p* < .001), and other opportunistic infections (20.5%, *p* = .008). Treatment-related factors associated with VF were first-line nevirapine-based regimen (27.7%, *p* = .009) and poor ART adherence (41.76%, *p* < .001).

**Conclusion:**

Sociodemographic factors, advanced HIV disease, co-morbidities, poor adherence, and specific first-line ART regimens are key predictors of virological failure. Targeted, multidisciplinary interventions focusing on routine viral load monitoring, adherence support, and addressing socioeconomic barriers are essential to improve ART outcomes in East Africa.

## Introduction

The widespread implementation of antiretroviral therapy (ART) has transformed the Human Immunodeficiency Virus/Acquired Immunodeficiency Syndrome (HIV/AIDS) landscape, significantly improving survival rates and quality of life for those living with the virus globally [1]. However, the long-term success of ART programs hinges on achieving and maintaining viral suppression among individuals on treatment. Virological failure (VF) defined as a plasma viral load above 1000 copies/mL after at least six months of ART, in the context of resource-limited settings, remains a persistent challenge that undermines the effectiveness of ART regimens [2].

In the context of East Africa, a subregion of sub-Saharan Africa disproportionately affected by the HIV/AIDS pandemic, monitoring and addressing VF is crucial for optimising treatment outcomes and preventing the emergence of drug resistance. Despite concerted efforts, several countries in this region continue to grapple with suboptimal viral suppression rates, falling short of the Joint United Nations Programme on HIV/AIDS (UNAIDS) 95–95–95 targets [3].

Previous systematic reviews and meta-analyses have attempted to quantify the burden of VF and its associated factors in sub-Saharan Africa. However, these studies have either focused on broader geographic regions or have become outdated due to the rapidly evolving landscape of HIV care and treatment, thus, necessitating a comprehensive and up-to-date synthesis specific to the East African context [4–6].

Failure to achieve and maintain viral suppression can lead to the accumulation of drug-resistant viral strains, increased risk of disease progression, and poor clinical outcomes [6,7]. Various studies conducted in sub-Saharan Africa have reported a wide range of VF prevalence, from 11% to 66%, among children and adolescents on ART [8–10]. However, the magnitude and predictors of VF in this population remain inadequately understood, particularly in the East African region. Through synthesising and analysing available data, this study seeks to enhance our understanding of the determinants of treatment failure and inform evidence-based interventions to mitigate the impact of VF on HIV treatment outcomes in the region.

This systematic review with meta-analysis and meta-regression aims at synthesising the available evidence on the incidence, predictors and prevalence of VF among persons living with HIV in the East African region. By systematically evaluating and synthesising evidence from published studies between 2016 and 2023, this review seeks to; estimate the pooled prevalence of VF across East African countries, identify and quantify the impact of key demographic, clinical, and programmatic factors associated with VF in this region, explore potential sources of heterogeneity across studies through meta-regression analyses and highlight knowledge gaps and areas requiring further research to inform evidence-based strategies for improving viral load monitoring and enhancing treatment outcomes.

The findings of this review provide a robust evidence base to guide policy decisions, resource allocation, and targeted interventions aimed at mitigating VF and optimising the long-term effectiveness of ART programs in East Africa. By addressing this critical challenge, the region can make strides towards achieving the UNAIDS targets and ultimately curbing the HIV/AIDS epidemic.

The period 2016 to 2023 is of interest as the World Health Organization (WHO) rolled out viral load monitoring as a standard measure for antiretroviral treatment response in 2014 and that informed the broader search of articles, however, most regions and countries in East Africa adapted and effected the guidelines in 2016 as reflected in the included studies [11].

## Materials and methods

### Systematic review protocol registration, information sources and search strategies

The aim of our systematic review and meta-analysis was intended to establish the prevalence and risk factors associated with VF among people with HIV enrolled on antiretroviral therapy (ART) in the United Nations (UN) East African region. The protocol was registered by International Prospective Register of Systematic Reviews (PROSPERO), University of York Center for Reviews and Dissemination (https://www.crd.york.ac.uk/PROSPERO), with a registration number CRD42023468918. Our results have been reported based on the conventional Preferred Reporting Items for Systematic Review and Meta-Analysis (PRISMA) 2020 statement checklist [12]. The following electronic databases including PubMed, Web of Science, ResearchGate, Scopus and African Journals Online (AJOL) were searched for primary records studies published from 2016 to 2023 to achieve our aim.

Three reviewers did a thorough review of the titles, abstracts and full papers. In case of any divergence in the views among the trio, this was resolved by agreement during the weekly evaluation meetings. In case of failure to agree, intervention of a senior person was done.

This meta-analysis used the Population, Exposure, Comparison and Outcome (PECO) strategy during the search strategy. For population, studies conducted on the VF for people with HIV on ART within the UN East African region were eligible for extracting data. For exposure status, the records that investigated exposed persons to HIV and on ART were eligible for data extraction. Regarding comparison, the studies that compared the predictors of suppression or VF (socio-demographic factors, clinical-related factors and treatment-related factors) among people living with HIV enrolled on ART were searched from the main data of the research article. Finally, for the outcome measure, the records that reported suppression or VF were used for data extraction in the main body of the article.

The search term used to retrieve the studies was (((Predictors) OR (Risk factors) OR (Determinants) OR (causes)) AND ((HIV) OR (Human Immunodeficiency Virus)) AND ((Virological failure) OR (Treatment failure) OR (Virologic failure)) AND ((East Africa) OR (Burundi) OR (Comoros) OR (Democratic Republic Congo) OR (Djibouti) OR (Ethiopia) OR (Eritrea) OR (Kenya) OR (Rwanda) OR (Seychelles) OR (Somalia) OR (Sudan) OR (South Sudan) OR (Tanzania) OR (Madagascar) OR (Uganda))) AND (2014:2024[pdat]). Filters applied from 2014 to 2024, performed in February 2024. After applying the inclusion/exclusion criteria, the final included studies were from 2016 to 2023. Key words: ‘Predictors’, ‘Prevalence’, ‘HIV’, ‘Virological failure’, ‘East Africa’. The key words were used with relevant MeSH terms (Medical Subject Headings) combined using Boolean operators ‘OR’ and ‘AND’.

### Eligibility criteria and study selection

Records included in the meta-analysis met the following criteria. First primary studies with full text data, cohort studies, case-control, randomised control trials and cross-sectional study designs that investigated the viral load among the people with HIV on ART. Second, those conducted among the UN East African member states https://www.afdb.org/en/countries/east-africa/east-africa-overview, Third, records published between 2016 to 2023 since the WHO rolled viral load monitoring in 2014 [11]. However, few countries adopted these guidelines immediately and publications were available from 2016. Finally, only records in English language were included. In contrast, studies published before 2023, in languages other than English, with inaccessible or insufficient data were excluded.

### Quality assessment and data management

For quality assessment, the Newcastle-Ottawa Scale (NOS) was used whereby studies with scores 9–8, 7–6 and 5–4 were considered to be of very high quality, high quality and moderate quality respectively. Studies with scores ≤3 were unsatisfactory and were rejected [13]. Quality assessment was independently done by three reviewers. The following variables have been extracted from each primary study; first author, year of publication, country, sample size, prevalence of virological failure, quality score (QS) and the predictors of VF.

### Risk of bias in individual studies

The bias evaluated was the selection bias for the primary records to be eligible for inclusion in the data synthesis. This was evaluated by reviewing the data collection procedures in the retrieved records (retrospective or prospective) and evaluating the study design (cohort, cross-sectional or case-control).

### Publication bias

For all the analyses, publication bias was assessed quantitatively and qualitatively. Quantitatively, Egger’s and Begg’s tests were used to evaluate the likelihood of publication bias. A *p* value >.05 indicated no evidence of publication bias. Qualitatively, the funnel plots were used to evaluate the potential of any publication bias. These were inspected for the symmetrical spread of the inverted funnel [14–16].

### Data synthesis

Throughout the meta-analysis, the random effect model (REM) or the fixed effect model (FEM) were used depending on the heterogeneity index (*I*^2^). For high heterogeneities among the primary studies, (*I*^2^ >50% and *P* het <0.05), the random-effects model (REM) meta-analysis was used. In contrast, when the heterogeneity was reduced (*I*^2^<50%, *P* het >0.05) the fixed-effects model (FEM) meta-analysis was used [17]. This was maintained when pooling the prevalence of virological failure, the predictors of virological failures and all the sub-group analyses. For all the analyses, the Medcalc software version 20.010 was used https://www.medcalc.org/manual/meta-analysis-introduction.php. The prevalence of VF or its predictor in each study was represented by the blue square in the forest plot whose size was indicative of the weight contributed by each study in the meta-analysis. The pooled prevalence of VF or its predictor for both FEM and REM were shown by the blue diamond.

## Results

### Study selection

**Figure 1.**
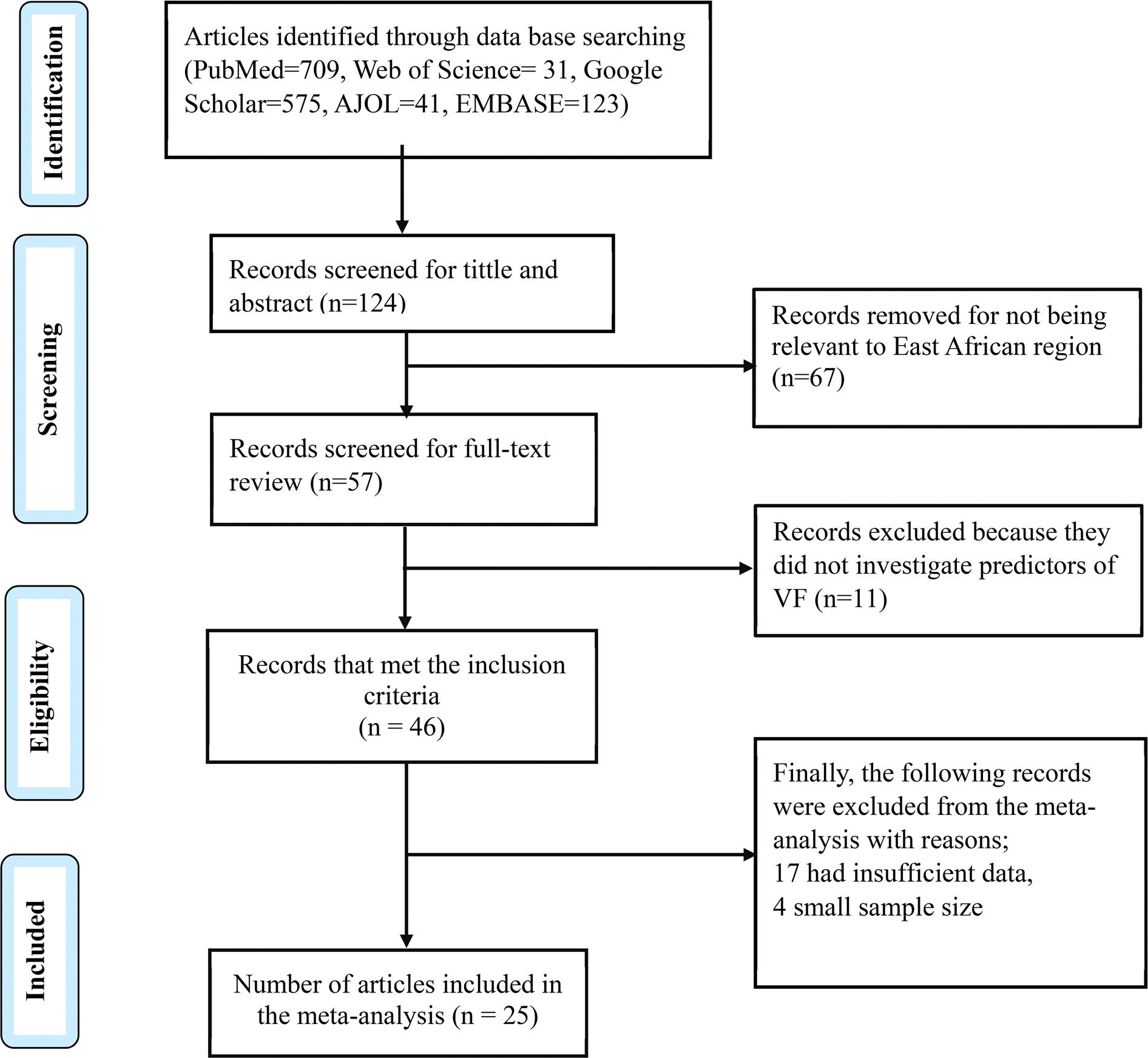
Flow chart for study eligibility following PRISMA criterion. AJOL: African journal online; VF: virological failure.

According to the PRISMA flow chat (Figure 1), we initially obtained 1479 articles through the primary database searching; 709 records from PubMed, 31 records from Web of Science, 575 records from Google Scholar, 123 from EMBASE and 41 from AJOL. Of these, 124 records were screened for titles and abstracts and 67 records were removed for not being relevant to the East African region leaving 57 records for screening for the full-text review. From these, 11 records were excluded because they did not investigate predictors of VF. Finally, 46 articles met the inclusion criteria from which the following studies were removed with reasons; 17 had insufficient data and 4 had inaccessible data. In total, 25 original studies were included in the data synthesis. The characteristics of each study have been presented in Table 1.

### The characteristics of the studies included in the meta-analysis

The characteristics of the eligible studies included in the meta-analysis are shown in Table 1. Briefly, of the 25 eligible studies for inclusion in the data synthesis. The majority of the studies were done in Ethiopia (12/25, 48.0%) with a total sample size of 4620; followed by Uganda (5/25, 20.0%) with a sample size of 2373; Tanzania (4/25, 16.0%) with a sample size of 3024; and Kenya (2/25, 8.0%%) with a sample size of 16,692; while Eretria and Rwanda had one eligible study each, with sample sizes of 1068 and 1688, respectively. Out of the 25 eligible studies, Kamau [37] had the largest sample size (of 16,340), whereas [25] had the smallest sample size (of 124) [25]. Most studies (11/25, 44.0%) with a total sample size of 7907 used were cohort studies, six studies (6/25, 24.0%) with a sample size of 2296 were case-control studies, six studies (6/25, 24.0%) with a sample size of 18,105 were cross-sectional studies, one study (1/25, 4.0%) with a sample size of 1169 was an observational study and one study (1/25, 4.0%) with a sample size of 352 was a randomised controlled trial (RCT). Finally, majority of the studies (9/25, 36.0%) with a sample size of 21,118 studied the prevalence of virological failure among adults, six studies (6/25, 24.0%) among the general population with a sample size of 2999, five studies (5/25, 20.0%) among children with a sample size of 1794, two (2/25, 8.0%) among adults and adolescents with a sample size of 826, two (2/25, 8.0%) among children and adolescents with a sample size of 689 and one (1/25, 4.0%) among sex workers with a sample size of 432.

### Pooled prevalence of virological failure in East Africa

**Figure 2.**
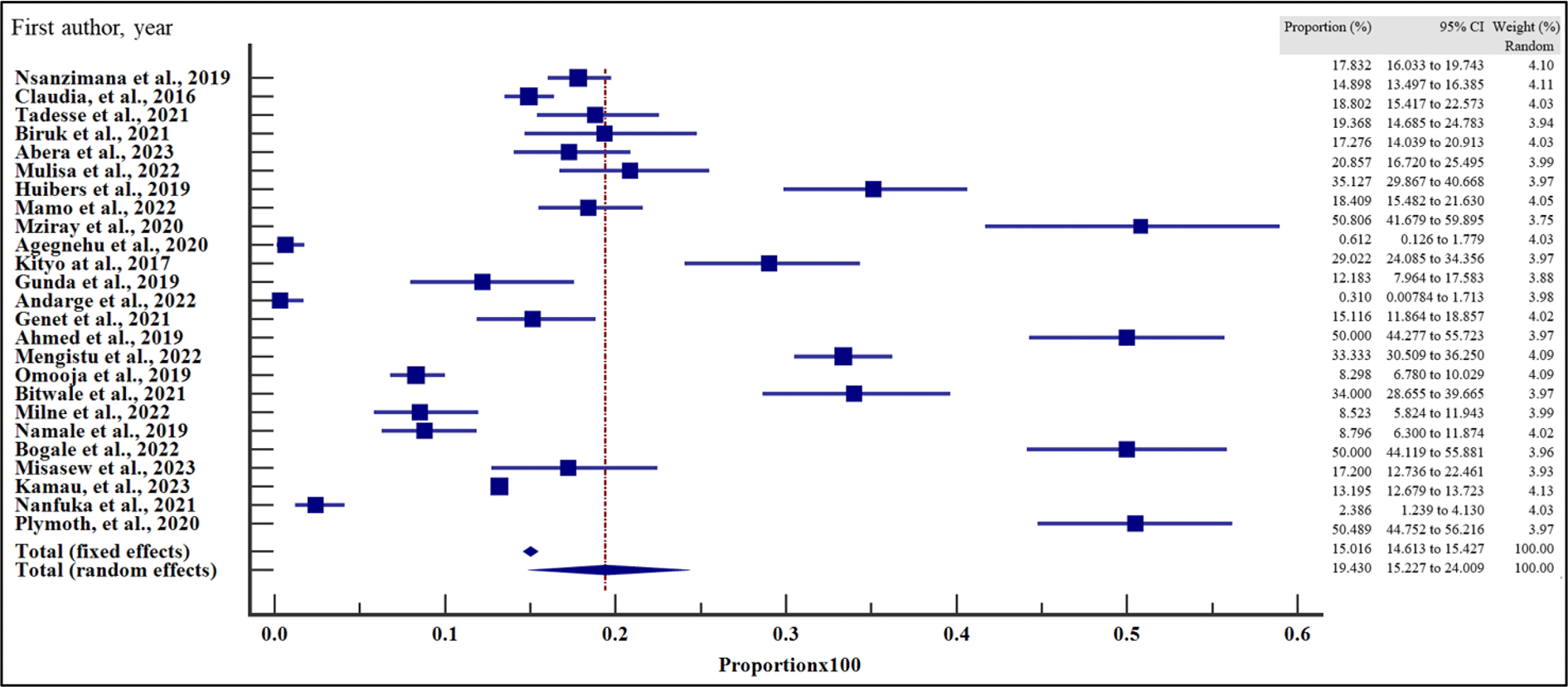
Pooled prevalence estimate of VF in East-Africa by random effects model.

**Figure 3.**
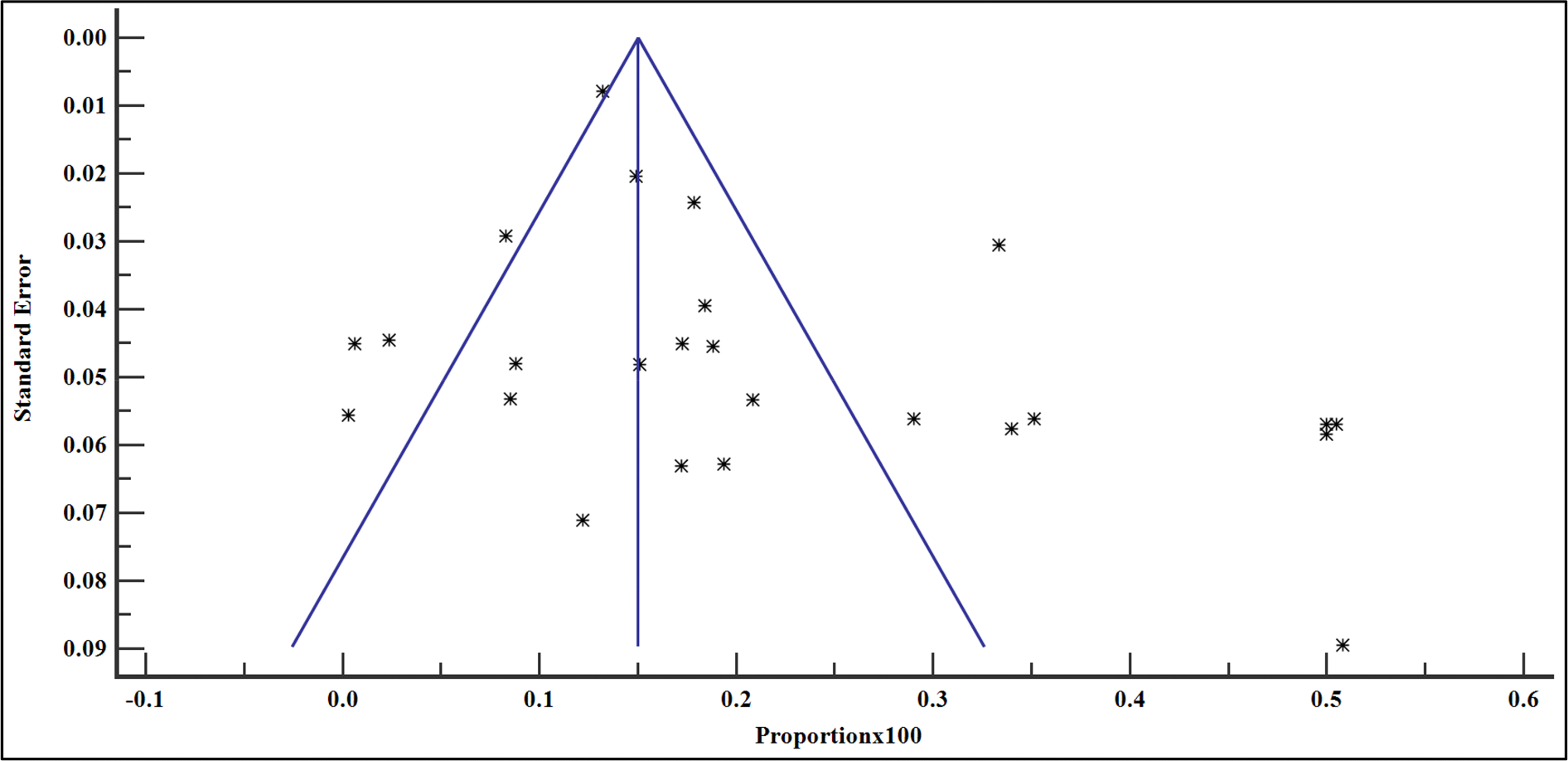
Bias assessment plot of studies reporting VF in East Africa from studies included in the data synthesis.

The prevalence of VF among the people with HIV on ART in East Africa between 2016 and 2023 varied, widely ranging from 0.31% (95% CI = 0.01 – 1.71%) reported in the study by Andarge et al. [28] in Ethiopia to 50.81% (95% CI = 41.68 – 59.90%) in another study reported by Mziray et al. [25] in Tanzania (Figure 2). The overall pooled prevalence of VF among the sample of 29,829 and 4723 cases was 19.40% (95% CI = 15.20 – 24.00% with a heterogeneity (*I*^2^) of 98.51% (*p* > .001) (Figure 3). The Egger’s test (*p* = .118) and Begg’s test (*p* = .076) demonstrated no evidence of publication bias among the analysis (Table 2). Similarly, funnel plot inspection for publication bias showed a symmetrical distribution of the studies suggesting no evidence of publication bias (Figure 3).

### Meta-analysis of the pooled prevalence of virological failure by sub-groups

As presented in Table 2, Our meta-analysis was sub-divided into sub-groups, which included the country where the study was conducted, age-groups and the year of publication. In all the sub-group analyses, the heterogeneity remained high (*I*^2^ > 60%, *p* < .05), so the random effect model (REM) was used for the analyses. Results showed that virological failure varied with the various sub-groups. For instance, by country, Tanzania had the highest prevalence of virological failure (26.5%, *p* < .001). By age groups, adults posted the overall highest prevalence of VF (32.7%, *p* < .001). Finally, previous publications, reported higher prevalence of virological failure than the recent years, (24.5%, *p* < .001).

### Meta-analysis of the factors associated with the prevalence of virological failure

#### Socio-demographic characteristics

Regarding the socio-demographic characteristics as predictors of VF, of the records retrieved from the data bases, 25 had disaggregated data on sex (Male or female); 14, on marital status (Married or not married); 11, on level of education (Primary and below or secondary and above); 8, on employment (formal employment or non-formal employment); 5, on HIV status disclosure (disclosed or not disclosed); 4, on location (rural or urban); 3, on the HIV status of the care giver (positive or negative). For all the analyses, the studies had very high heterogeniety (*I*^2^ > 50%, phet < 0.001) and hence the random effects model was used for the meta-analysis. In addition, there was no evidence of publication bias for the eligible studies for the analysis of the predictors of virological failure when evaluated by both Egger’s and Beggs tests except for the analysis of gender (Table 3).

The results of our meta-analysis have shown that the following factors were associated with higher chance of having VF:: - First, the men were more likely to have experienced VF than women (30.90% vs 26.20%, *p* < .001) Second, being un married was more likely to have experienced VF than being married (28.25% vs 19.32%, (*p* < .001). Third, having attained a lower level of education at primary level or lower at a prevalence of 32.97% (95% CI = 19.22 – 48.39%) compared to those who reported to have attained secondary education and above at 25.67% (95% CI = 16.76 – 35.75) (*p* < .001). Fourth, having non-formal employment at a prevalence of 47.16% (95%CI = 28.90 – 65.83% compared to those with formal employment at a prevalence of 22.59% (95% CI = 14.67 – 31.66%) (*p* < .001). Fifth, non-status disclosure at a prevalence of 32.55% (95%CI = 7.18 – 65.50%) as compared to those who disclosed their status at a prevalence of 20.6% (95% CI = 9.88 – 34.07%) (*p* < .001). Sixth, being an urban dweller at a prevalence of 51.16% (95% CI = 8.9 – 92.33% as opposed to the rural dweller (*p* < .001). And finally, the HIV status of parent/caregiver with HIV having a higher prevalence of virological failure at a prevalence of 32.79% (95% CI = 10.78 – 59.86% as opposed to those whose care givers are HIV negative at a prevalence of 23.19% (95% CI = 8.47 – 42.43) (*p* = .012). For all the analyses, the heterogeneity remained high (*I*^2^ > 88%, *p* < .05) and hence the random effect model was used for the analysis.

#### Clinical-related characteristics

Pertaining to the clinical-related characteristics as predictors of VF, of the records retrieved from the data bases, 5 had disaggregated data on functional status (working or ambulatory); 4, on body mass index (underweight or normal); 13, on CD4 T-cell count (low or normal); 5, on haemoglobin concentration (anaemic or normal); 11, on WHO HIV staging (stage I/II or stage III/IV); 6, on TB (HIV/TB co-infection or HIV mono-infection); 6, on other opportunistic infections (positive or negative). Consistent with the analysis of the socio-demographic factors as predictors of virological failure, the studies had very high heterogeniety (*I*^2^ > 50%, phet < 0.001) and hence the random effects model was used for the meta-analysis. Furthermore, there was no evidence of publication bias for the eligible studies for the analysis of the predictors of virological failure when evaluated by both Egger’s and Beggs tests (Table 4).

Interesting results have been obtained from our data synthesis on the clinical-related characteristics associated with virological failure. The prevalence of HIV virological failure was significantly associated with the several indicators: - First, being ambulatory at a prevalence of 44.70% (95% CI = 29.30 – 60.50%) (*p* < .001) from a pooled sample size of 342 participants. Second, having a low CD4 T-cell count at a prevalence of 35.10% (95% CI = 25.10 – 45.80%) (*p* < .001) from a pooled sample size of 2975. Third, having a low haemoglobin concentration at 52.20% (95% CI = 37.90 – 66.30%) (*p* < .001) from a pooled sample of 402 participants with HIV. Fourth, being at WHO HIV stage III/IV at a prevalence of 44.20% (95% CI = 30.40 – 58.40%) (*p* < 0.001) from a pooled sample size of 1806. Fifth, having a history of HIV/TB co-infection at a virological failure prevalence rate of 24.30% (95% CI = 17.00 – 32.60%) (*p* < .001) from a pooled sample of 557 participants. Lastly, having a history of other opportunistic infections at a prevalence rate of VF of 20.50% (95% CI = 12.70 – 29.50%) (*p* = .008) from a pooled sample size of 792.

Again, for all the analyses, the heterogeneity remained high (*I*^2^ > 75%, *p* < .05) and hence the random effect model was used for the analysis.

#### Treatment-related characteristics

Regarding the treatment-related factors as predictors of VF, of the records retrieved from the data bases, 9 had disaggregated data on first line HIV treatment regimen as efavirenz-based (EFV-based) or nevirapine-based (NVP-based), 6 on regimen change (yes or no), 2 on second-line treatment as LPV-based or ATV-based and 19 on adherence to treatment as good or poor. For all the analyses, the heterogeneity remained high (*I*^2^ > 85%, *p* < .05) and hence the random effect model (REM) was used to pool the prevalence of virological failure during the analysis. However, there was no heterogeneity for the second-line regimen and the (*I*^2^ = 0.00%, *p* > .05) and the fixed effect model (FEM) of meta-analysis was used (Table 5).

Our data synthesis on the treatment-related characteristics associated with VF has shown that VF was significantly associated with first-line nevirapine-based regimen with a prevalence of 27.70% (95% CI = 17.80 – 38.70%) (*p* = .009) from 9 studies with a pooled sample size of 1471. Similarly, poor adherence to treatment at a prevalence of 41.76% (95% CI = 32.90 – 50.90%) from 19 studies with a pooled sample size of 2077 was significantly associated with VF (*p* < .001). Other treatment-related factors including regimen change and second-line regimens were not significantly associated with VF (*p* > .05).

#### Factors associated with the risk of virological failure

To investigate the relative risk of virological failure, we meta-analysed the data on the statistically significant factors from socio-demographic, clinical- and treatment-related factors. These are presented in Tables 6–8.

#### Socio-demographic factors

**Figure 4.**
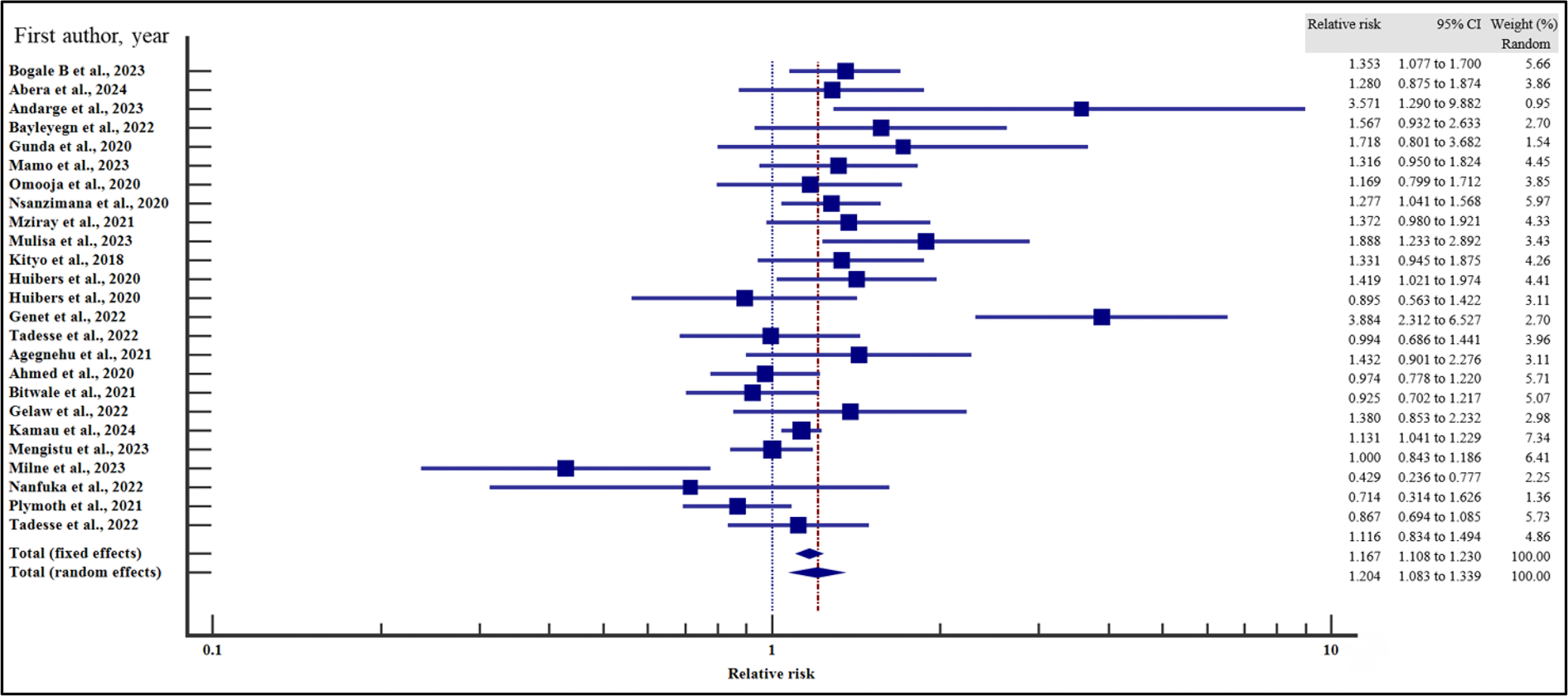
Sex and the risk of VF when compared between the male and the female. The RR > 1 indicates increased risk of VF whereas the RR < 1 indicates reduced risk of VF.

**Figure 5.**
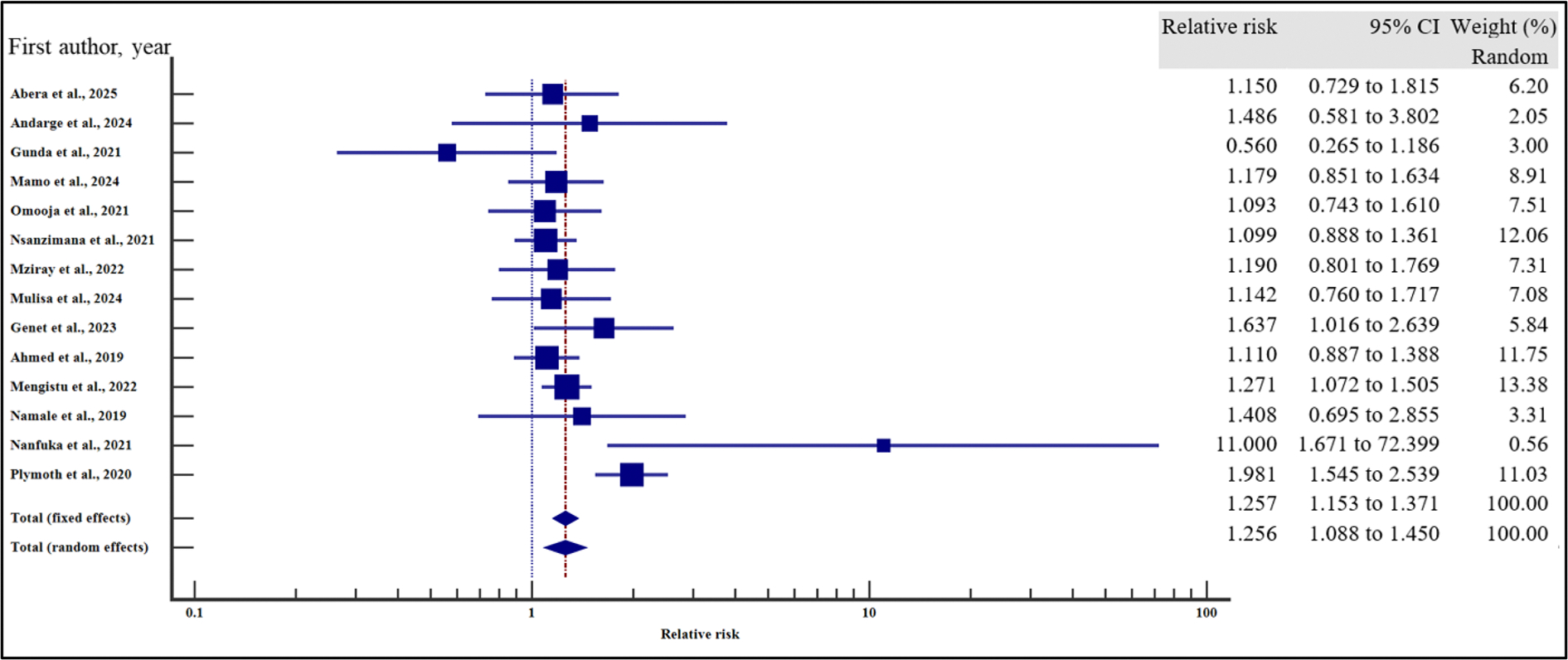
Marital status and the risk of VF when compared between the male and the female. The RR > 1 indicates increased risk of VF whereas the RR < 1 indicates reduced risk of VF.

**Figure 6.**
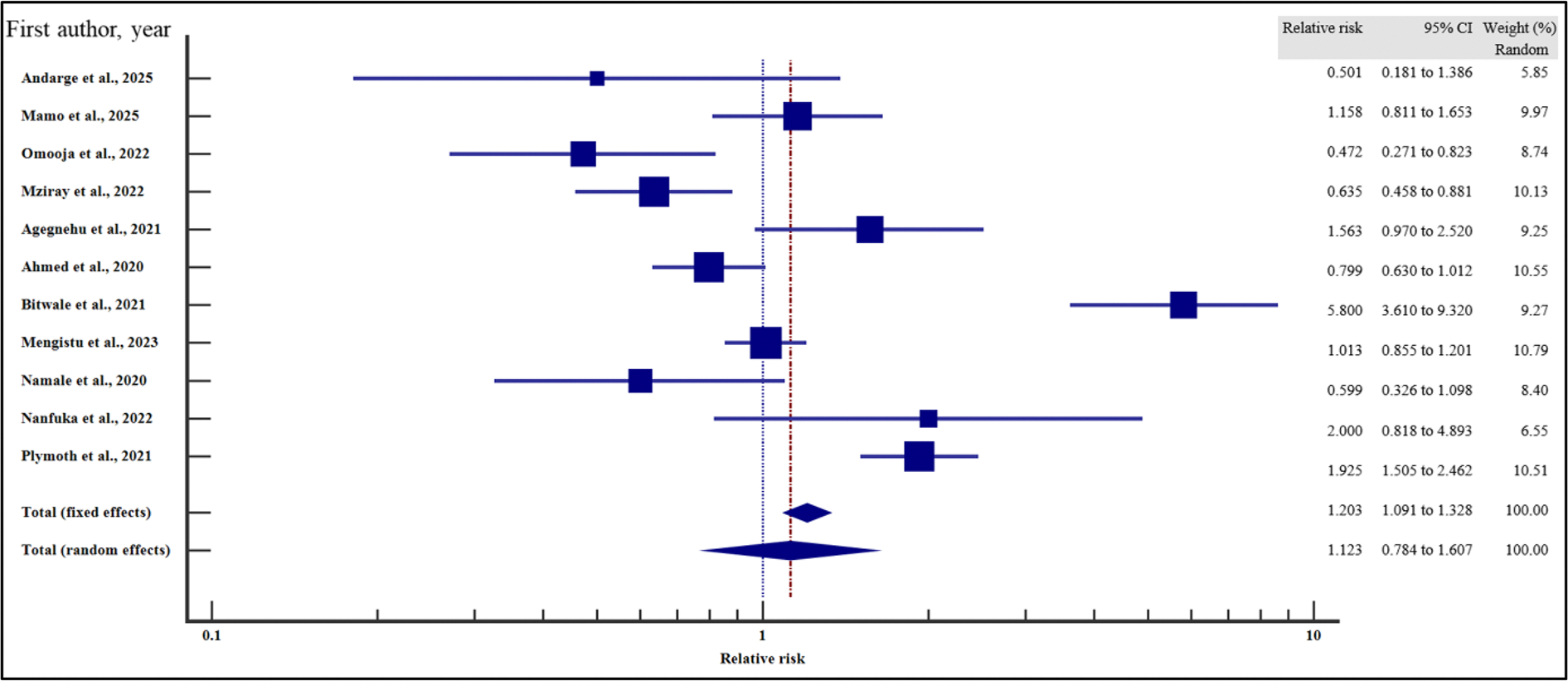
Level of education and the risk of VF when compared between those who attained primary or lower level of education and those who attained secondary or higher. The RR > 1 indicates increased risk of VF whereas the RR < 1 indicates reduced risk of VF.

**Figure 7.**
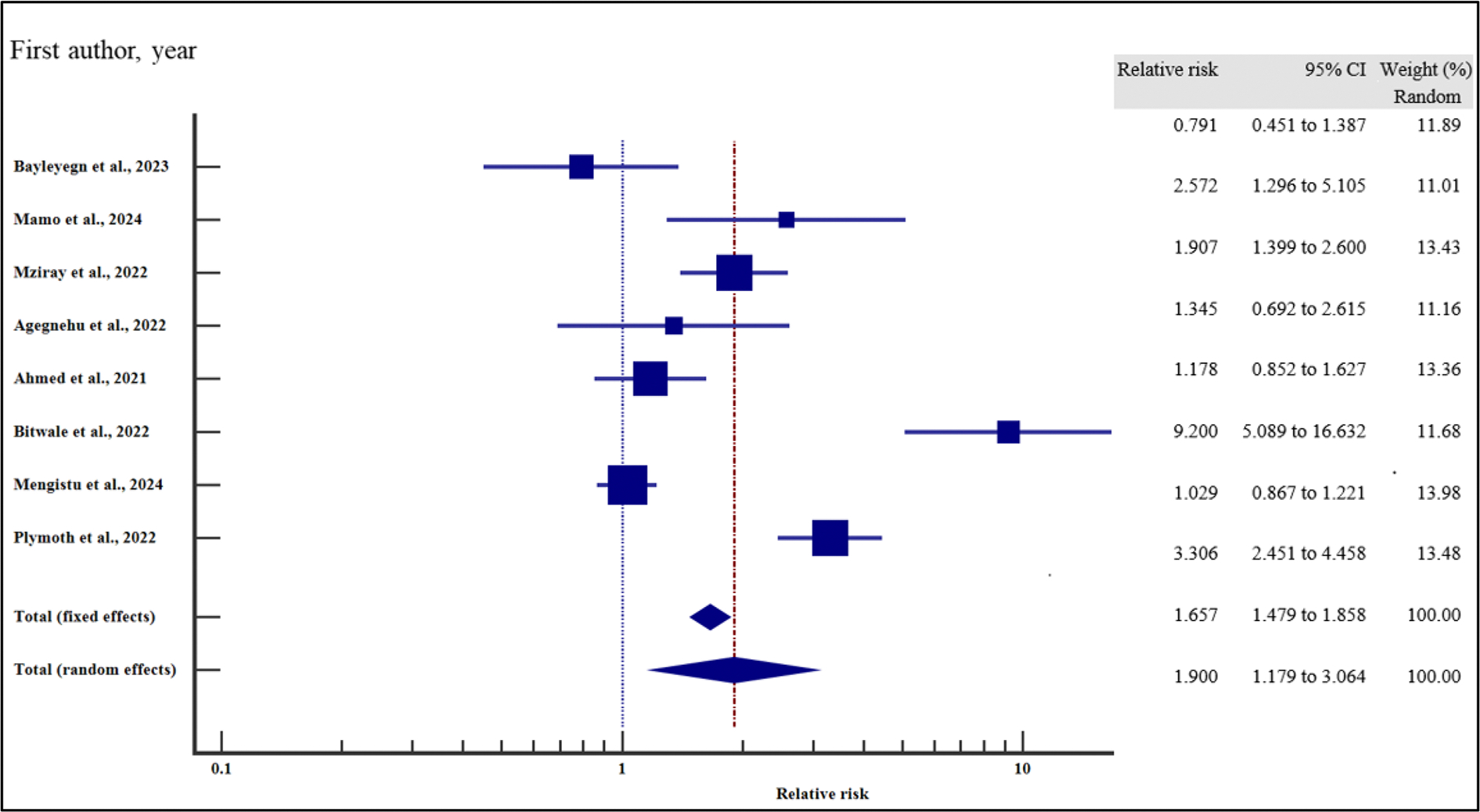
Employment and the risk of VF when compared between those with formal and non-formal employment. The RR > 1 indicates increased risk of VF whereas the RR < 1 indicates reduced risk of VF.

**Figure 8.**
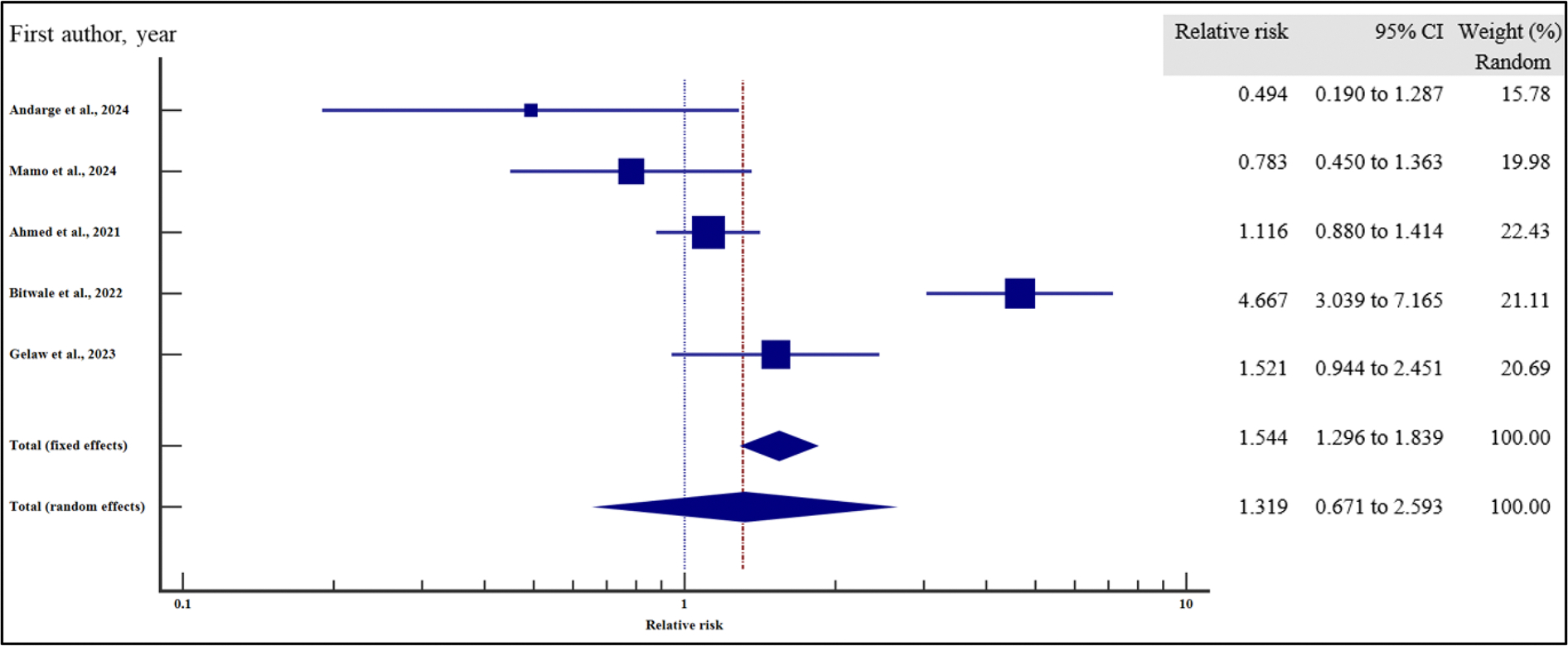
HIV status disclosure and the risk of VF when compared between those who disclosed their HIV status and those who did not disclose. The RR > 1 indicates increased risk of VF whereas the RR < 1 indicates reduced risk of VF.

**Figure 9.**
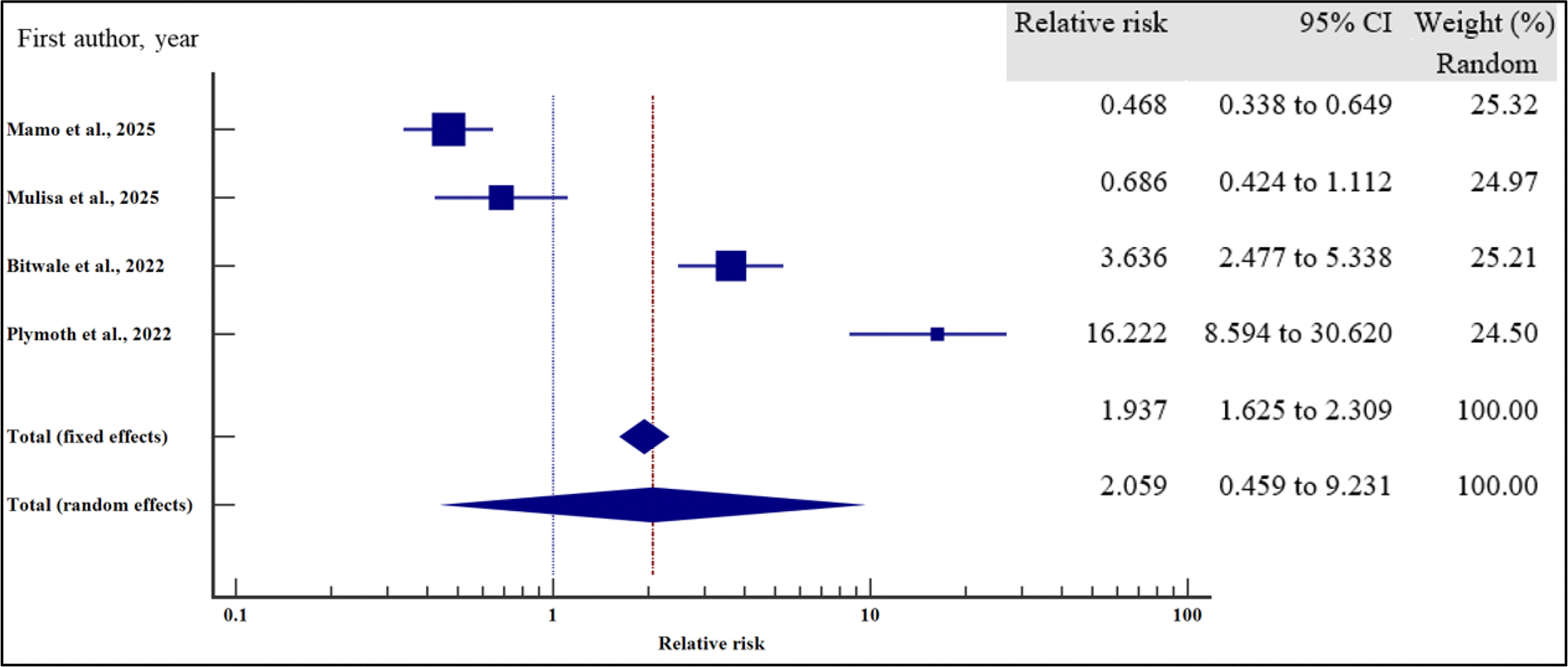
Location and the risk of VF when compared between rural and urban study participants. The RR > 1 indicates increased risk of VF whereas the RR < 1 indicates reduced risk of VF.

**Figure 10.**
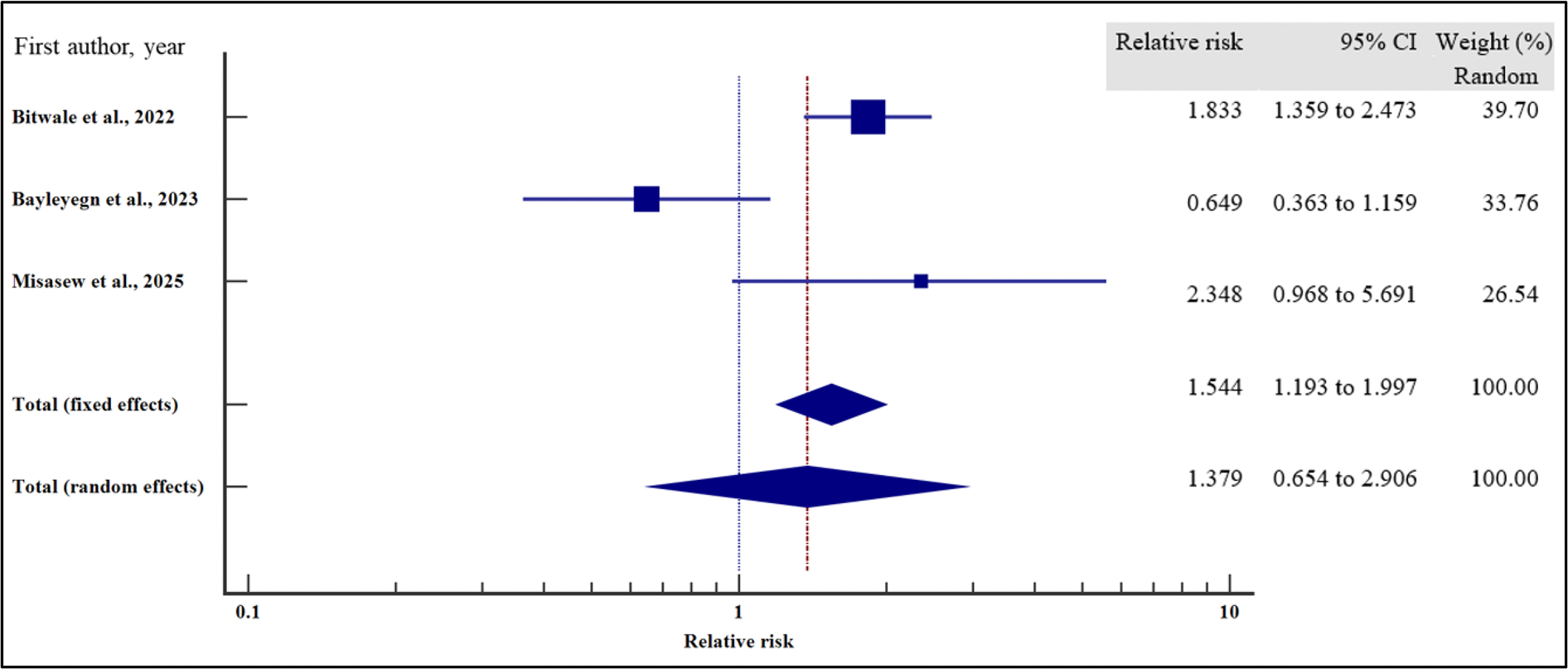
HIV status of the caregiver/parent and the risk of VF when compared between care givers with HIV and care givers with negative HIV status. The RR > 1 indicates increased risk of VF whereas the RR < 1 indicates reduced risk of VF.

In order to evaluate the effect of the socio-demographic characteristics on the risk of VF, we performed a meta-analysis using relative risk as the effect measure. As presented in Figures 4–10 and Table 6, being a male increased the risk of VF 1.2 times compared to a female counterpart on ART (CI =1.08 – 1.34%, *p* = .001). Similarly, being unmarried increased the risk of VF by 1.26 times as opposed to being married (95% CI = 1.09 to 1.45%, *p* = .002). Finally, having a non-formal employment by the people with HIV or their care givers increased the risk of non-suppression by almost two times compared to those persons with HIV or their care givers with a formal employment (95% CI = 1.18 – 3.06%, *p* = .008).

In contrast, level of education (relative risk 1.12, (95% CI = 0.78 – 1.61%, *p* = .528), HIV disclosure status (relative risk 1.32, 95% CI = 0.67 – 2.59%, *p* = .423), location (relative 2.06, 95% CI = 0.46 – 9.23%, *p* = .345) and HIV status of the care giver (relative risk 1.38, 95% CI = 0.65 – 2.91%, *p* = .40) where not significantly associated with the risk of VF.

For all the analyses, the heterogeneity index (*I*^2^) remained high (≥ 50%, *p* het < 0.05) and hence the random effect model was used for the meta-analysis. Similarly, when the records were evaluated for publication bias, there was no evidence of publication bias by both Beggs and Egger’s tests (*p* > .05).

#### Clinical-related characteristics and the risk of VF

**Figure 11.**
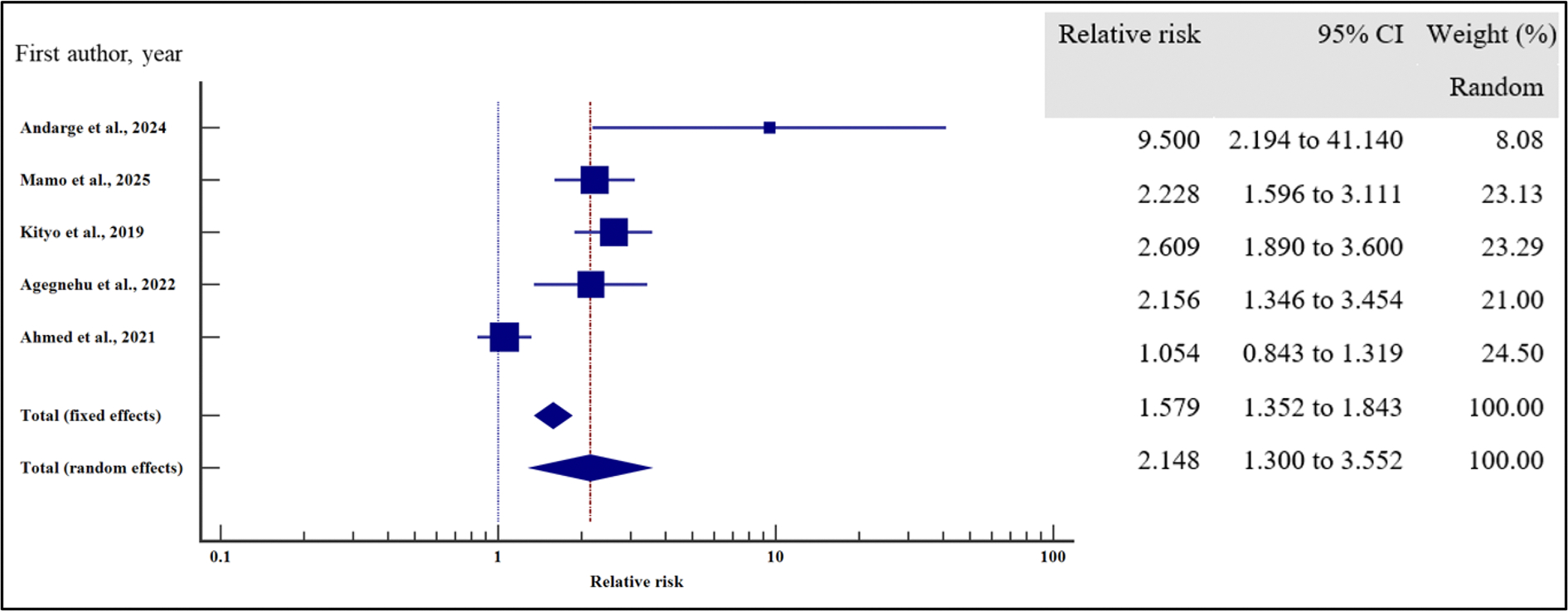
Functional status and the risk of VF when compared between rural and urban study participants. The RR > 1 indicates increased risk of VF whereas the RR < 1 indicates reduced risk of VF.

**Figure 12.**
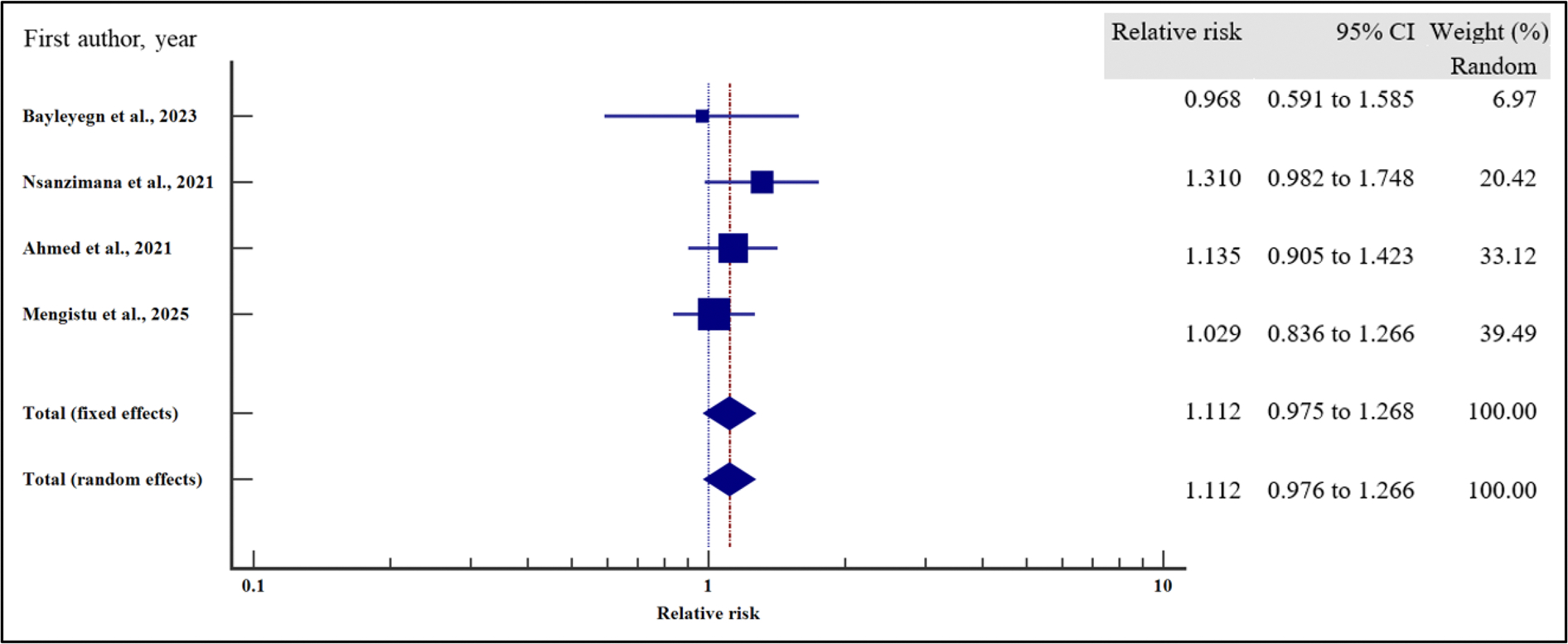
Body mass index and the risk of VF when compared between rural and urban study participants. The RR > 1 indicates increased risk of VF whereas the RR < 1 indicates reduced risk of VF.

**Figure 13.**
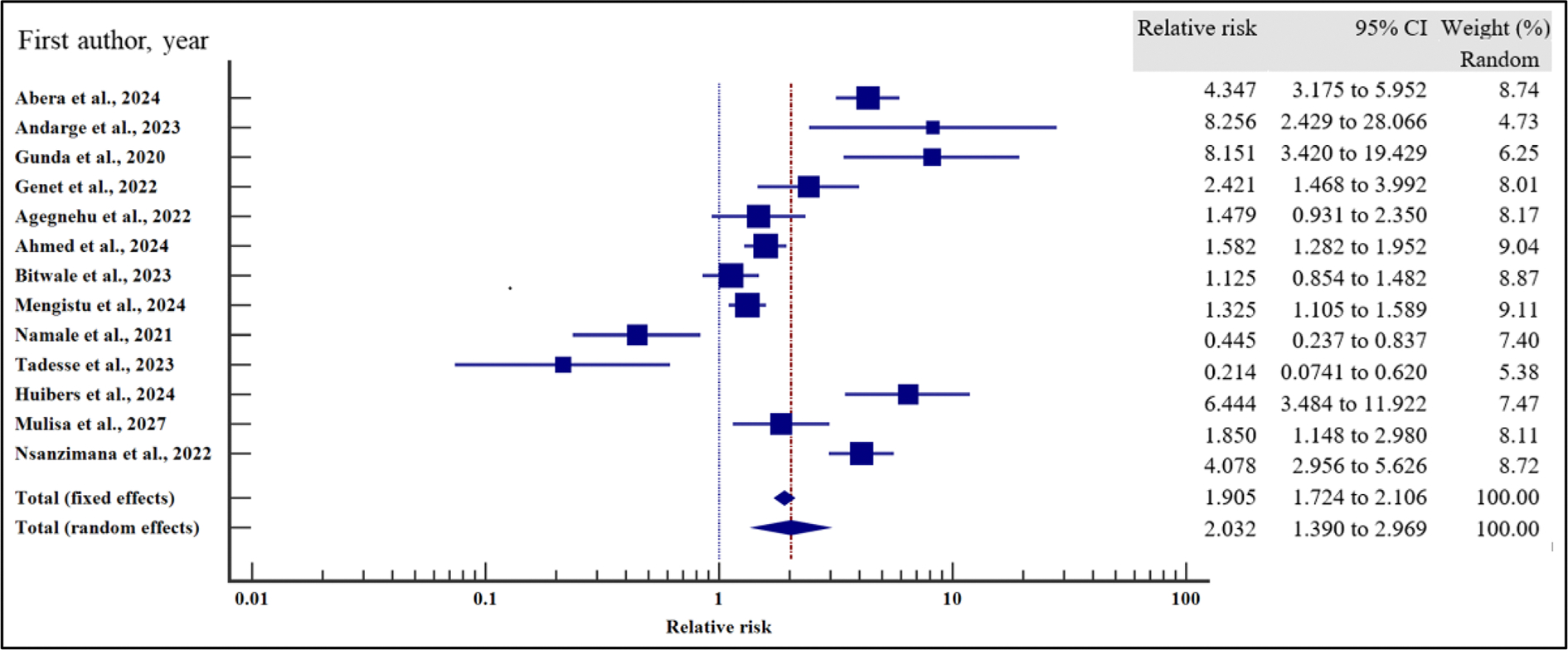
CD4 Count and the risk of VF when compared between rural and urban study participants. The RR > 1 indicates increased risk of VF whereas the RR < 1 indicates reduced risk of VF.

**Figure 14.**
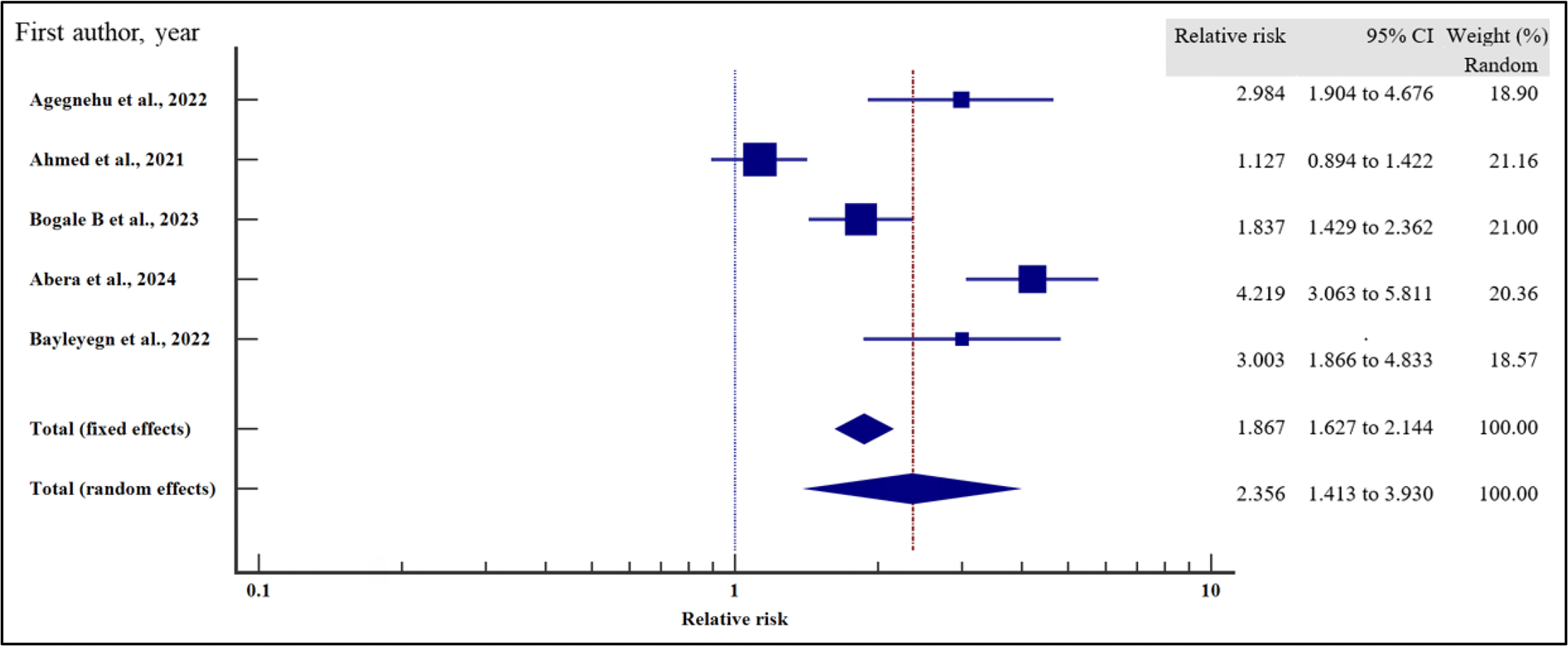
Anaemia and the risk of VF when compared between rural and urban study participants. The RR > 1 indicates increased risk of VF whereas the RR < 1 indicates reduced risk of VF.

**Figure 15.**
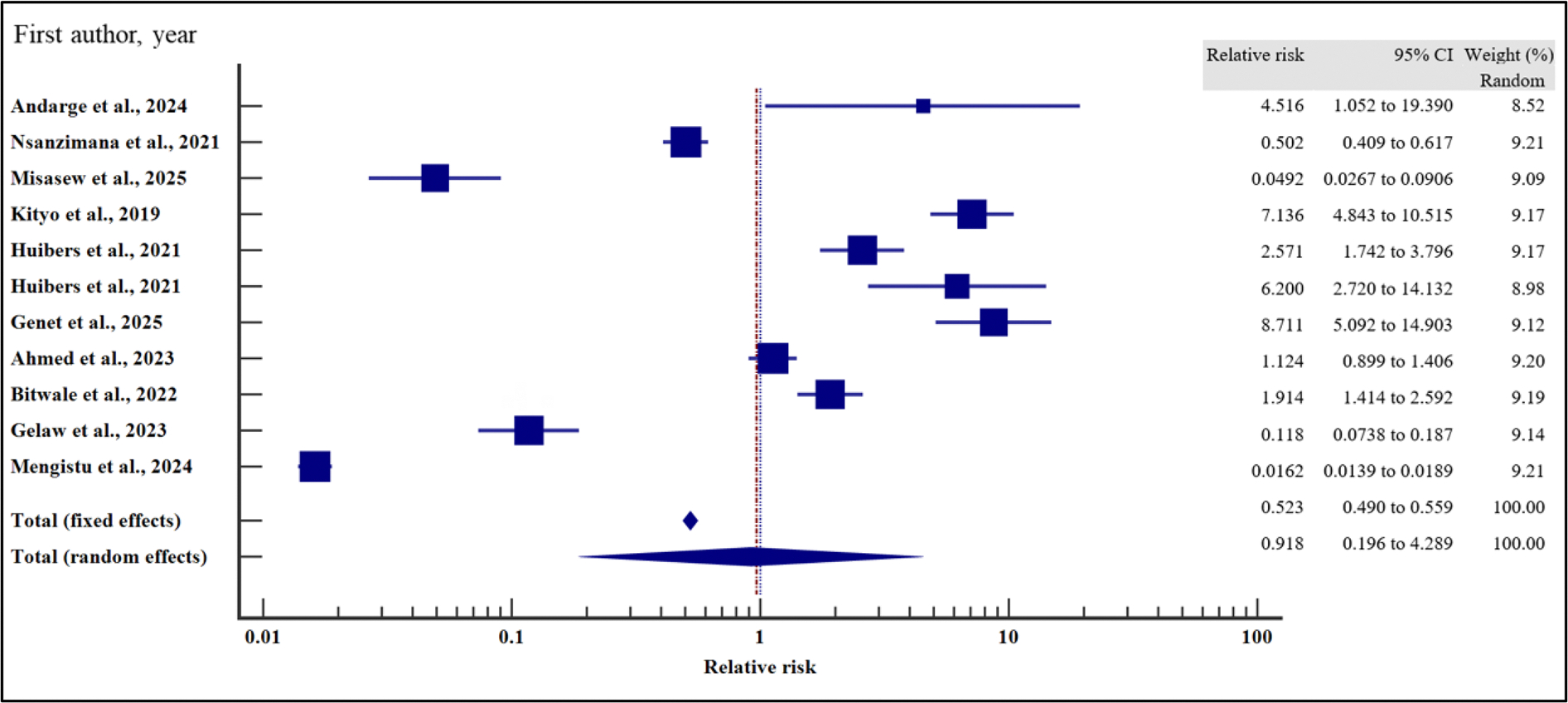
WHO HIV staging and the risk of VF when compared between rural and urban study participants. The RR > 1 indicates increased risk of VF whereas the RR < 1 indicates reduced risk of VF.

**Figure 16.**
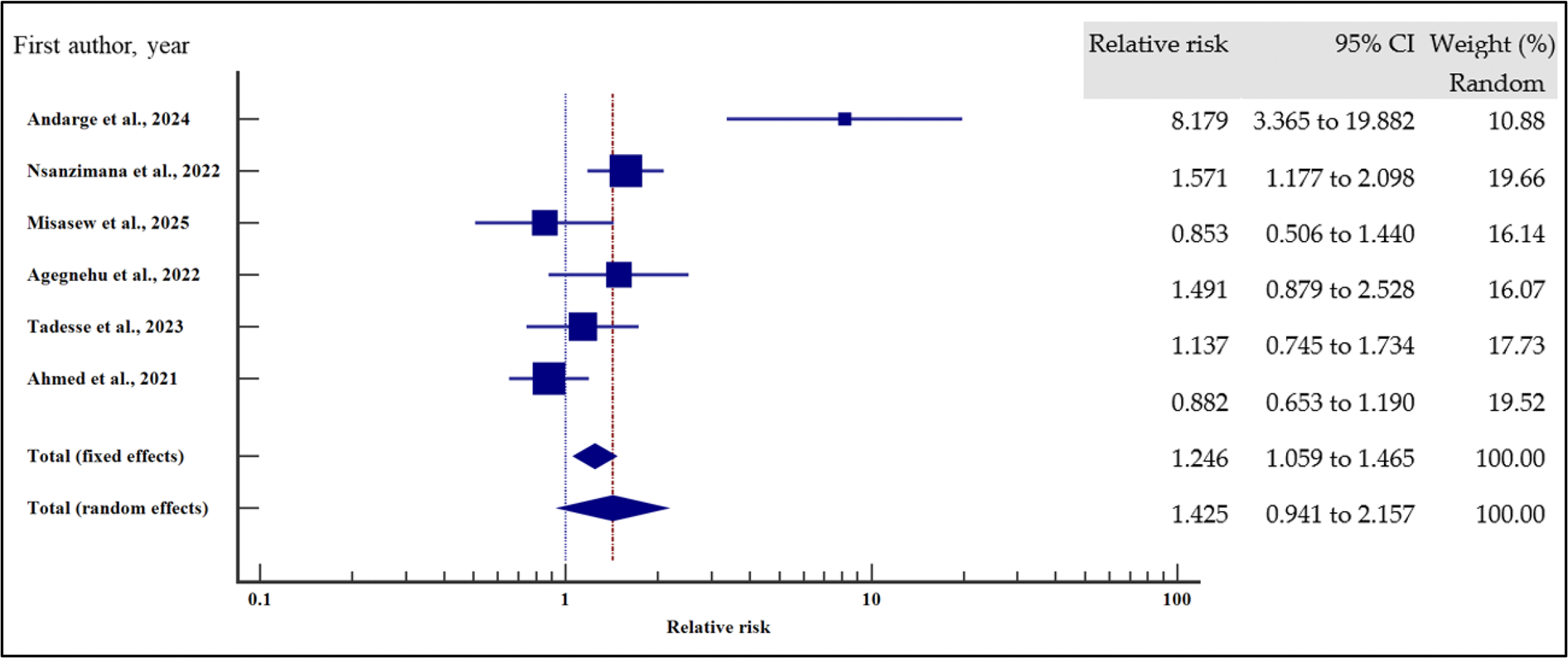
HIV/TB co-infection and the risk of VF when compared between rural and urban study participants. The RR > 1 indicates increased risk of VF whereas the RR < 1 indicates reduced risk of VF.

**Figure 17.**
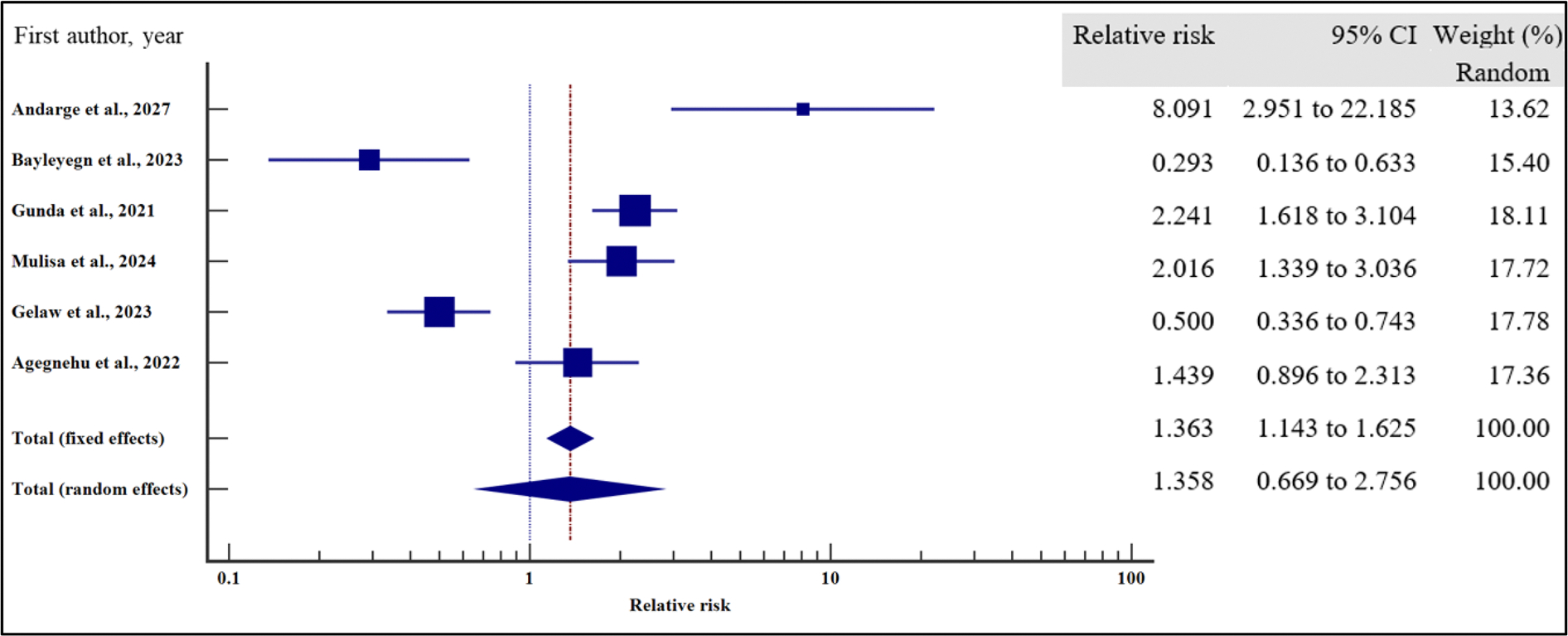
HIV co-infection with other opportunistic infections and the risk of VF when compared between rural and urban study participants. The RR > 1 indicates increased risk of VF whereas the RR < 1 indicates reduced risk of VF.

Furthermore, we evaluated the influence of clinical characteristics on the risk of virological failure with HIV using relative risk meta-analysis as the effect measure. As presented in Figures 11–17 and Table 7, being ambulatory at the time of enrolment on ART increased the risk of VF by two times compared to those who were fully active at the point of enrolment (95% CI = 1.30 – 3.55%, *p* = .003). Similarly, a low CD4 T-cell count at the time of recruitment on ART increased the risk of virological non suppression by two times (95% CI = 1.39 – 2.97%, *p* < .001). Finally, the risk of VF was two times higher if the patients were enrolled on ART when anaemic as opposed to those with normal haemoglobin concentration (95%0. CI =1.41 – 3.93%, *p* = .001).

On the other hand, body mass index (relative risk 1.11, 95% CI = 0.98 – 1.27%, *p* = .115), WHO HIV staging (relative risk 92, 95% CI = 0.20 – 4.29%, *p* = .913), HIV/TB co-infection (relative risk 1.43, 95%CI = 0.94 – 2.16%, *p* = .094) and infection with other opportunistic infections other than TB (relative risk 1.36, 95% CI = 0.67 – 2.76%, *p* = .397) at the time of enrolment were not significantly associated with VF.

For all the analyses, the heterogeneity index (*I*^2^) remained high (*I*^2^ ≥ 50%, *p* het < 0.05) and the random effect model was used to pool the relative risk. However, the heterogeneity index (*I*^2^) was reduced for body mass index (*I*^2^ < 50%, *p* het > 0.05) and the random effect model was used to pool the relative risk. Interestingly, when we evaluated the potential publication bias for the published records used to pool the relative risk during our data synthesis, there was no evidence of publication bias by both Beggs and Egger’s tests (*p* > .05).

#### Treatment-related characteristics and the risk of VF

We also evaluated the influence of treatment-related factors on the risk of VF among people with HIV receiving ART.

**Figure 18.**
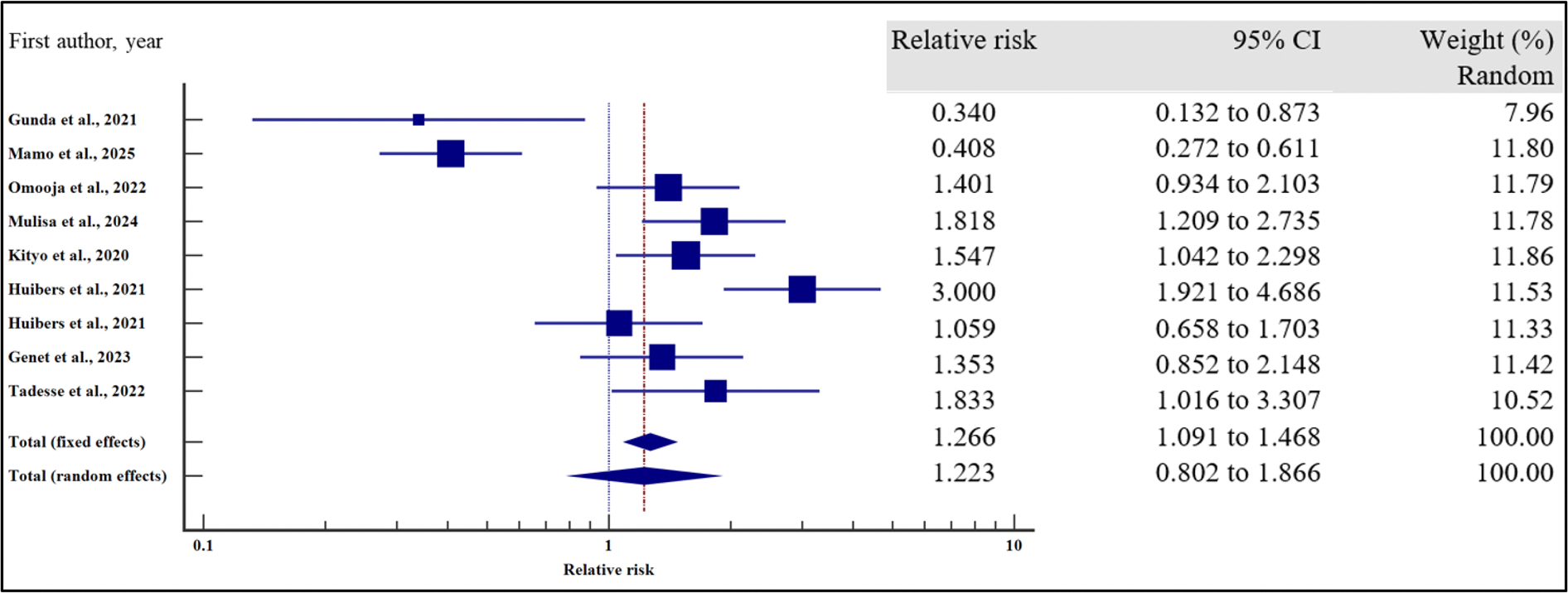
EFV/NVP treatment regimen and the risk of VF when compared between rural and urban study participants. The RR > 1 indicates increased risk of VF with NVP whereas the RR < 1 indicates reduced risk of VF with NVP.

**Figure 19.**
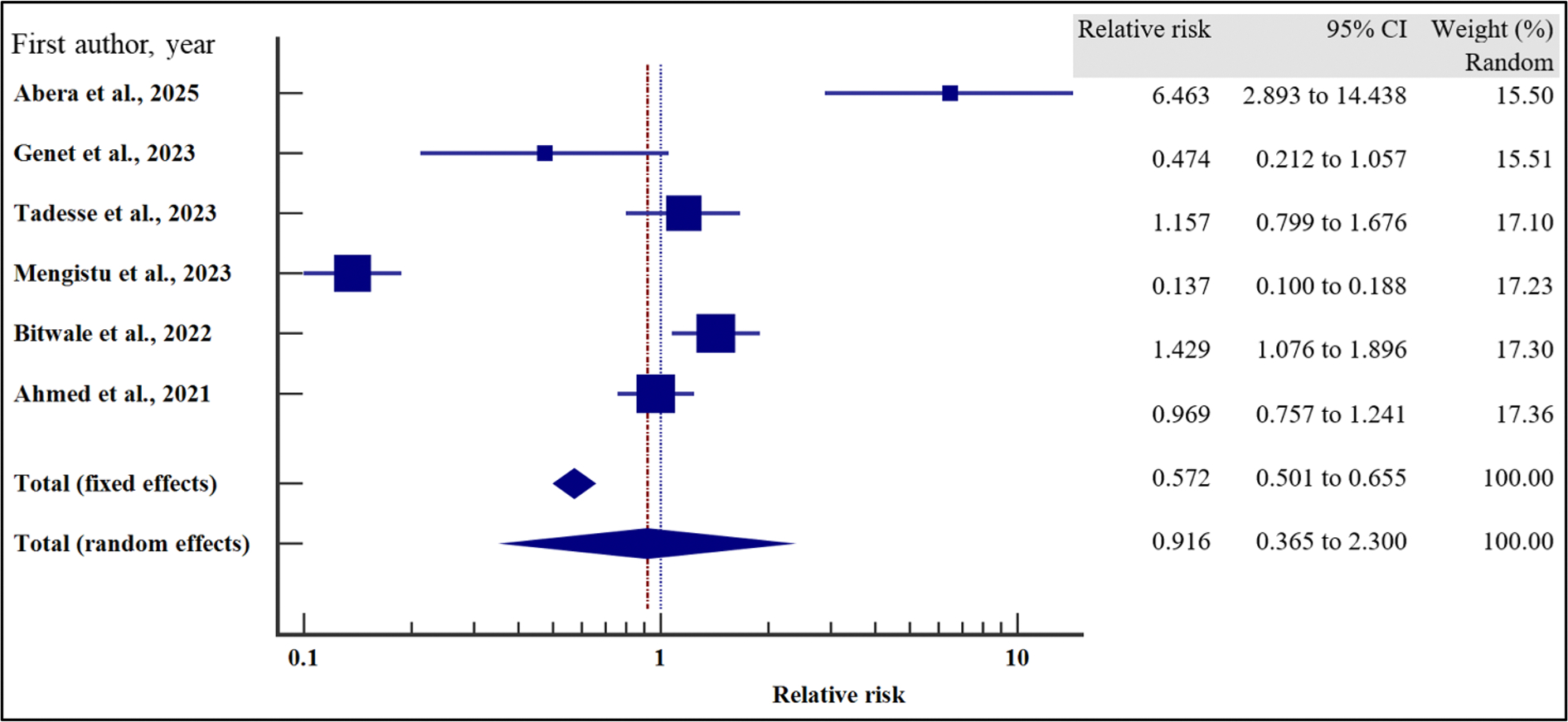
Change of treatment regimen and the risk of VF when compared between rural and urban study participants. The RR > 1 indicates increased risk of VF whereas the RR < 1 indicates reduced risk of VF.

**Figure 20.**
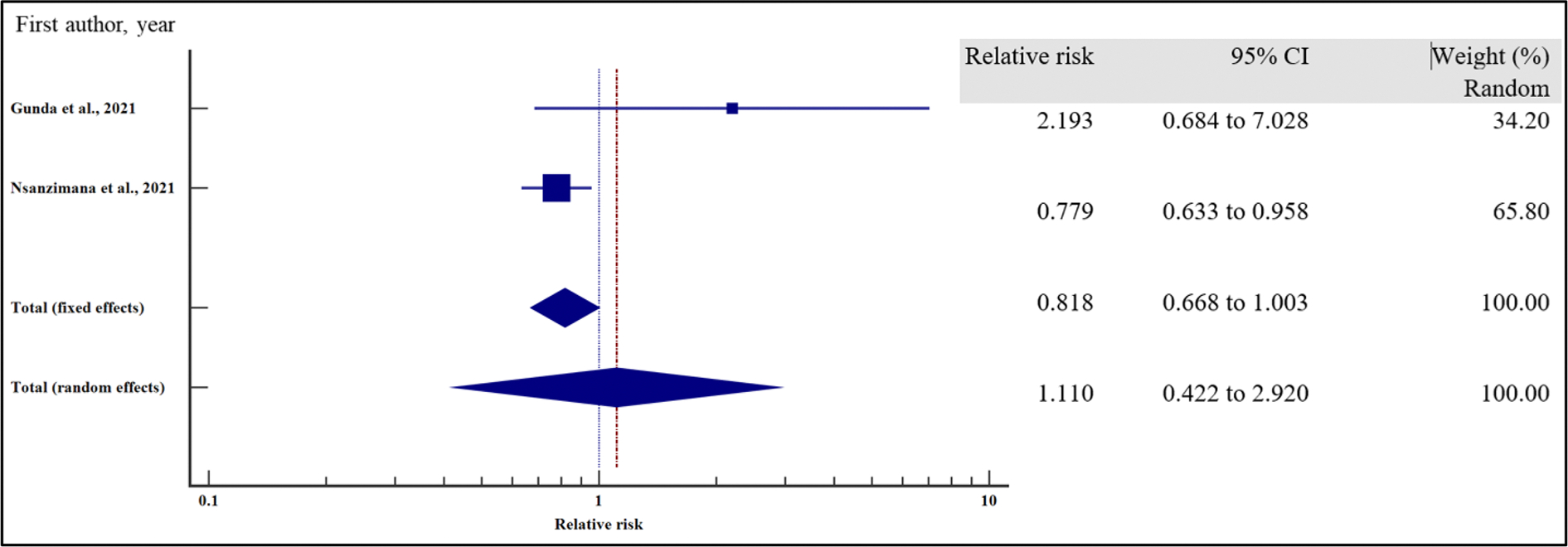
LPV/ATV treatment regimen and the risk of VF when compared between rural and urban study participants. The RR > 1 indicates increased risk of VF with LPV whereas the RR < 1 indicates reduced risk of VF with LPV.

**Figure 21.**
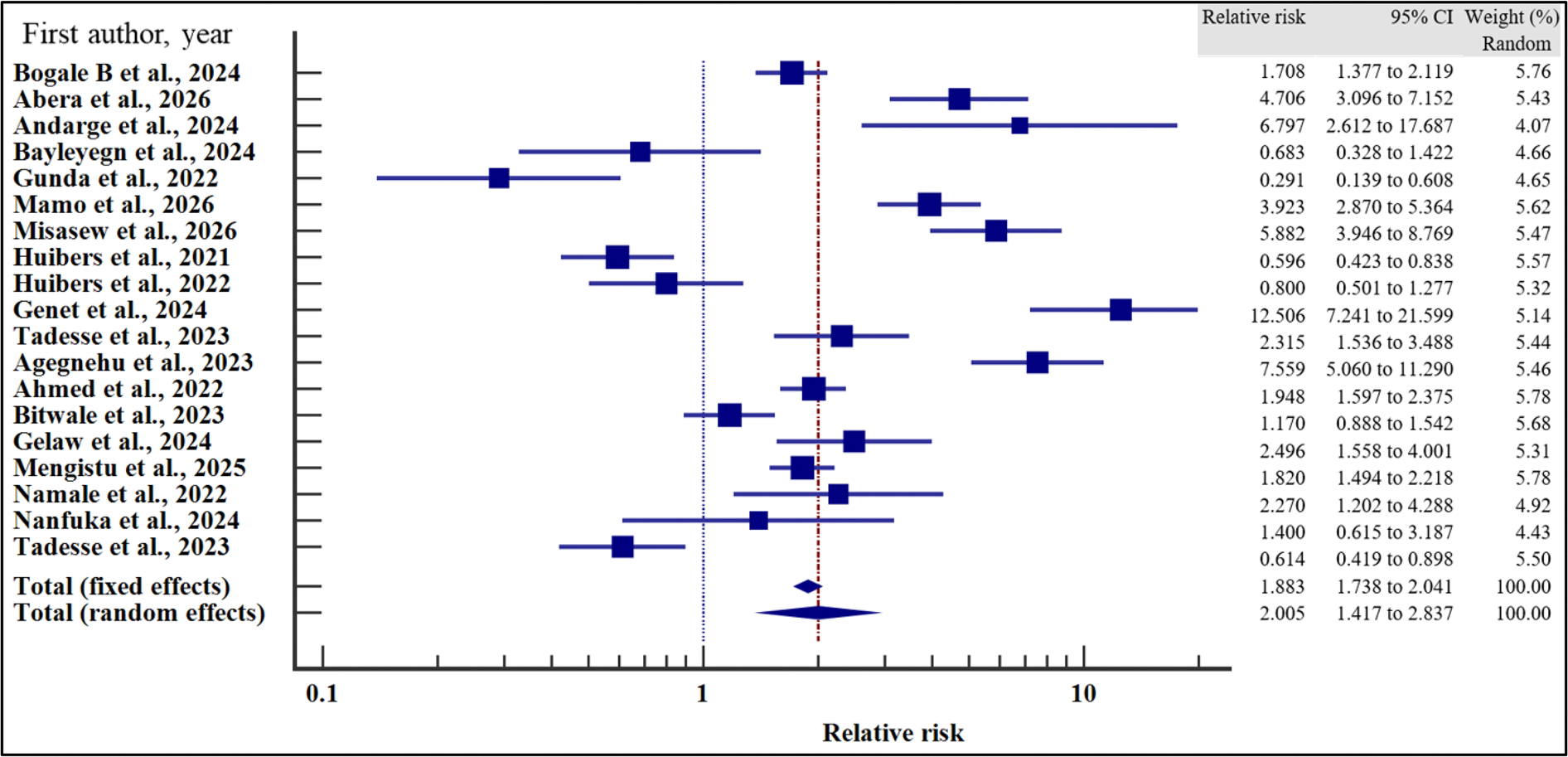
Adherence and the risk of VF when compared between rural and urban study participants. The RR > 1 indicates increased risk of VF whereas the RR < 1 indicates reduced risk of VF.

As presented in Figures 18–21 and Table 8, our results have shown that poor adherence (relative risk 2.01, 95% CI = 1.42 – 2.84%, *p* < .001) was the only independent risk factors significantly associated with virological non suppression. Thus, poor adherence on ART increased the risk of VF by two times compared to those with good adherence. Other treatment-related factors including regimen (first line or second line) and regimen change were not risk factors significantly associated with VF (*p* > .05).

For all the analyses, the heterogeneity index (*I*^2^) remained high (*I*^2^ > 66%, *p* het < 0.05) and the random effect model was used to pool the relative risk.

### Meta-regression analysis for the overall variation in the VF, by country and study groups between 2016 and 2023

**Figure 22.**
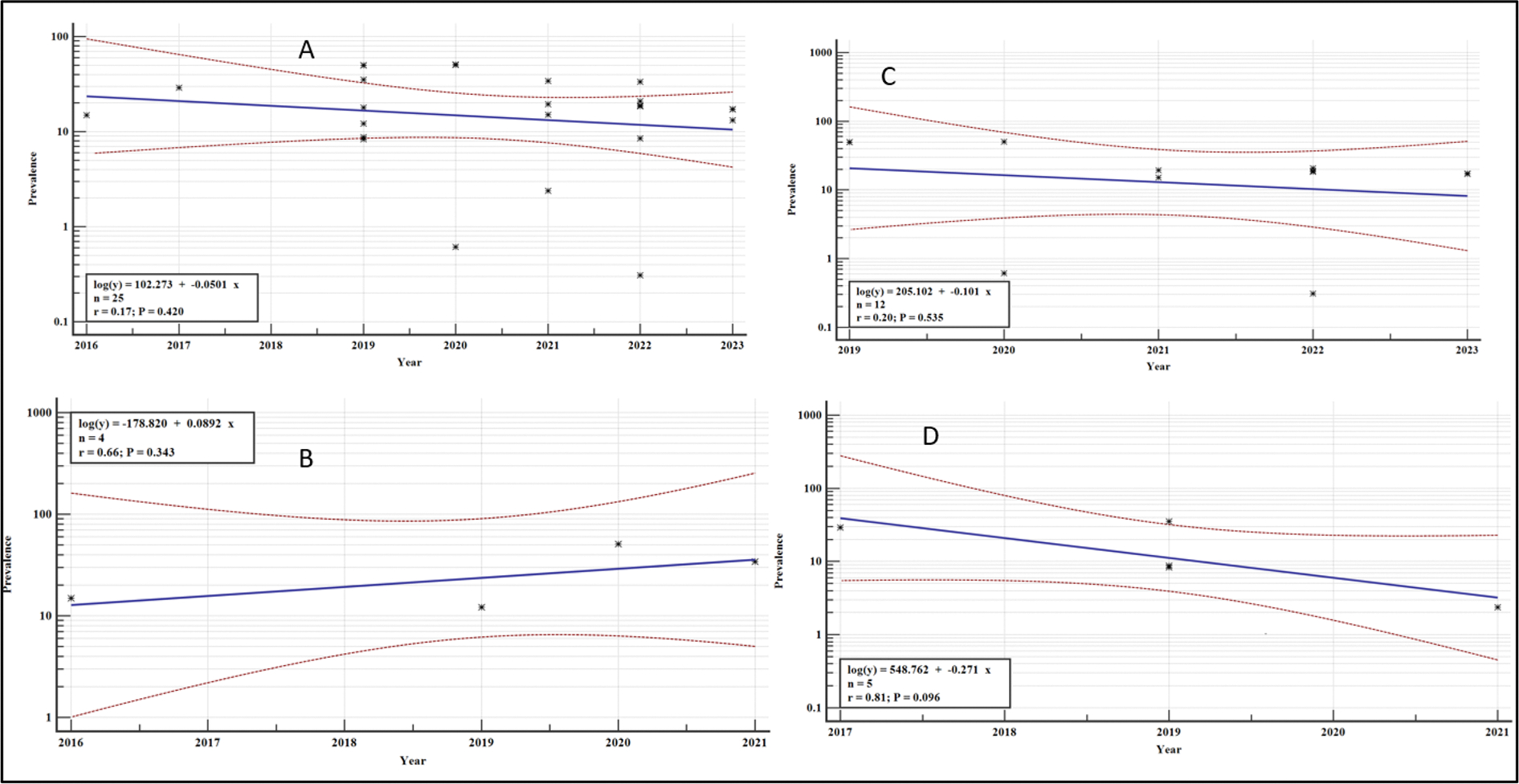
Meta-regression analysis of the percentage prevalence of VF: A: Overall, B: Tanzania, C: Ethiopia and D: Uganda.

**Figure 23.**
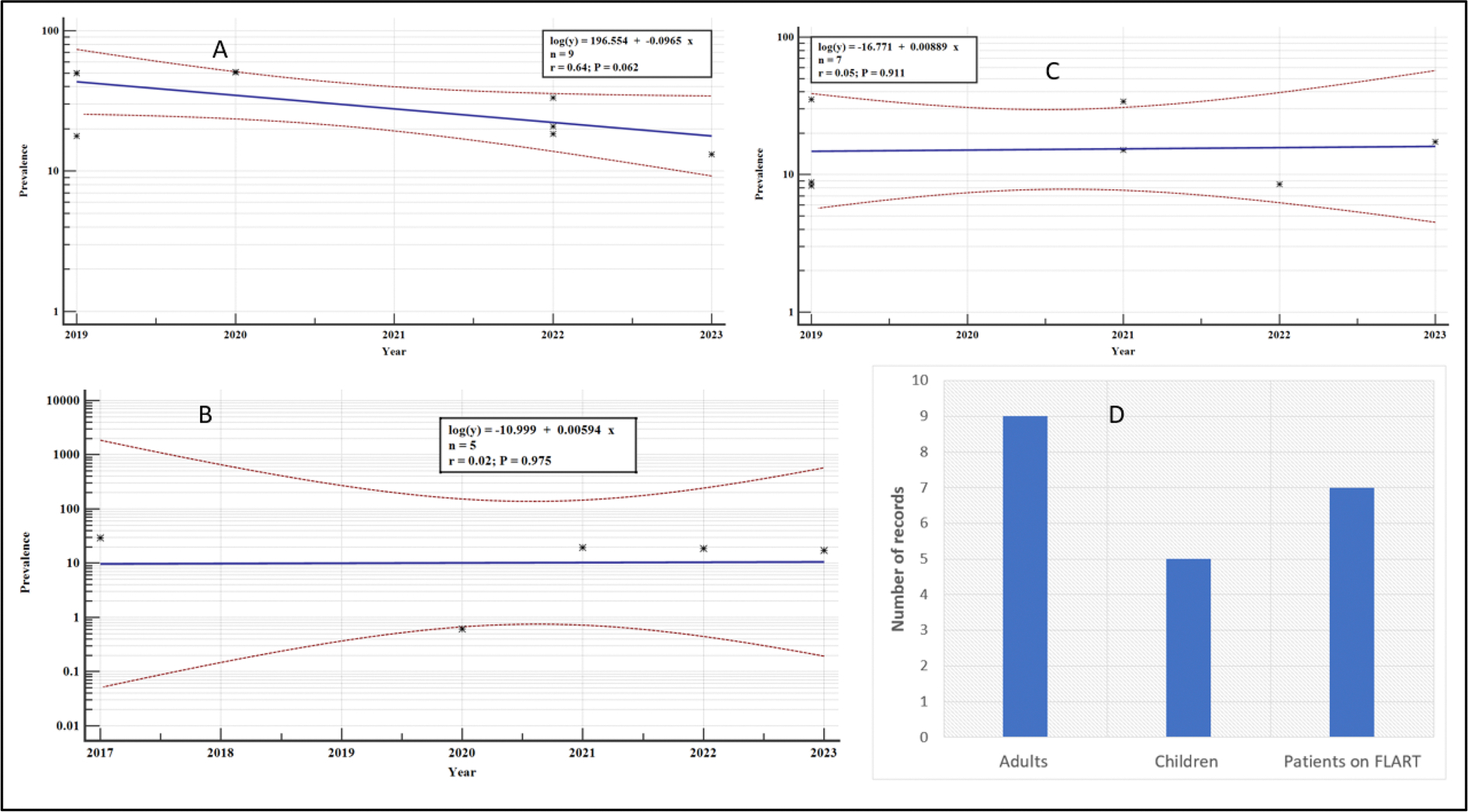
Meta-regression analysis of the percentage prevalence of VF: A: Adults, B: Children, C: Patients on FLART, D: Number of records for each study group.

We performed a meta-regression to give constructive clues on the variations in the prevalence of VF over the years of study in order to guide future research on the management of VF among the people with HIV on ART in our region. The results are shown in Figures 22 and 23. The variations in the overall pooled prevalence of VF and the pooled prevalence by sub group analysis did not differ significantly (*p* > .05). However, there was a general decline in the overall prevalence of VF over the years (regression equations: *log (y)*= −*0.0501x* +*102.273)*, for Ethiopia (regression equations: *log (y)*= −*0.101x* +*205.102)* and for Uganda: (regression equations: *log (y)*= −*0.271x* +*548.762)*. In contrast, studies from Tanzania, reported a general increase in VF over the years (regression equation: *log (y)*= +*0.0892x* −*178.820)* (Figure 22).

In addition, the variations in the prevalence of VF for the study group over the years did not differ significantly (*p* > .05). However, there was a general decline in the prevalence of VF among adults (regression equations: *log (y)*= −*0.0965x* + *196.554*) but an increase in the prevalence of VF among the children (regression equations: *log (y)*= +*0.00549x* −*10.999*) and persons on FLART (regression equations: *log (y)*= +*0.00889x* −*16.771*) (Figure 23).

### Sensitivity analysis

**Figure 24.**
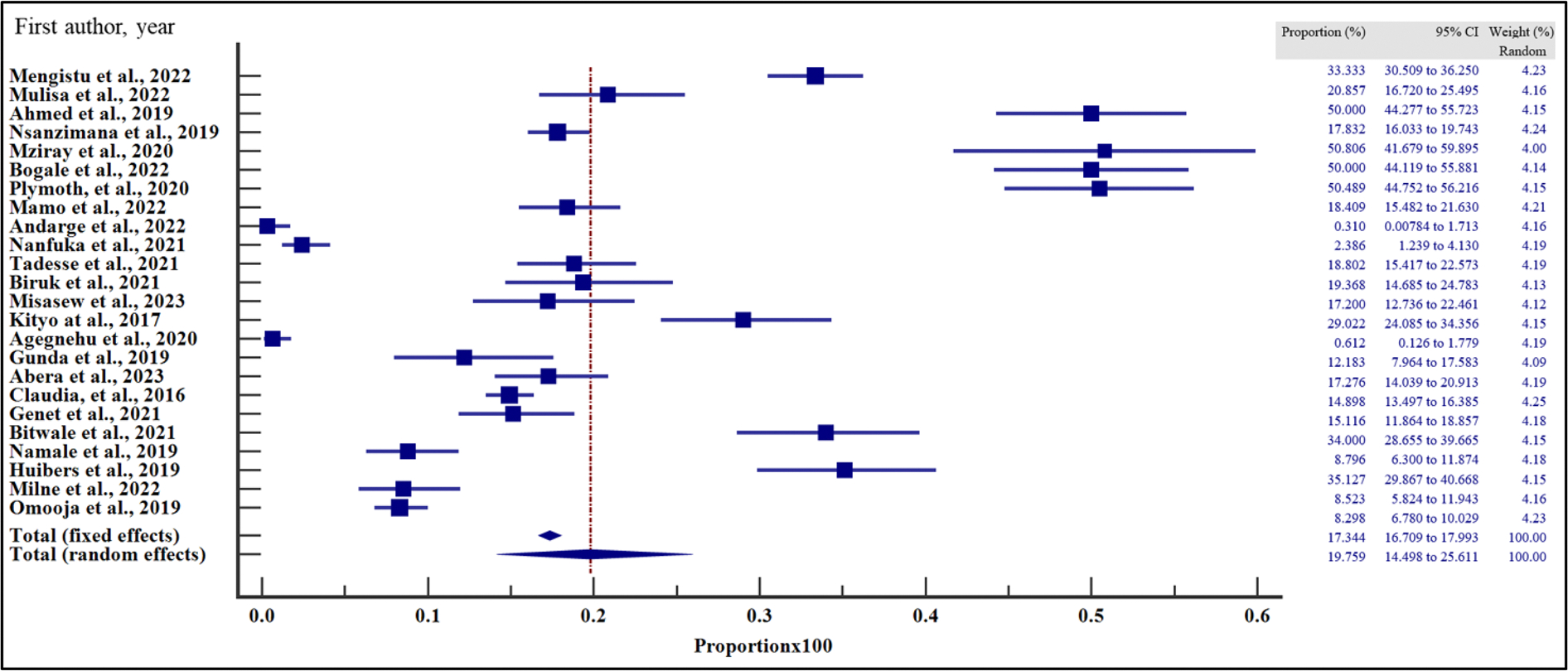
Forest plot for sensitivity analysis with one study with the highest sample size omitted.

We performed a sensitivity analysis by removing the study with the largest sample size reported by Kamau (37]. The pooled prevalence before omission was 19.4% (95% CI =15.20 – 24.00%) with a heterogeneity (*I*^2^) of 98.51%, *p* > .001. After the omission, the pooled prevalence increased slightly to 19.8% (95%CI = 14.50 – 25.60%) with a heterogeneity (*I*^2^) of 98.48%, *p* < .001 (Table 2, Figure 24). This suggests that the pooled prevalence was not affected by a single study. Besides, the pooled prevalence of VF did not differ significantly by single study omission (*p* > .05) (Table 2).

**Figure 25.**
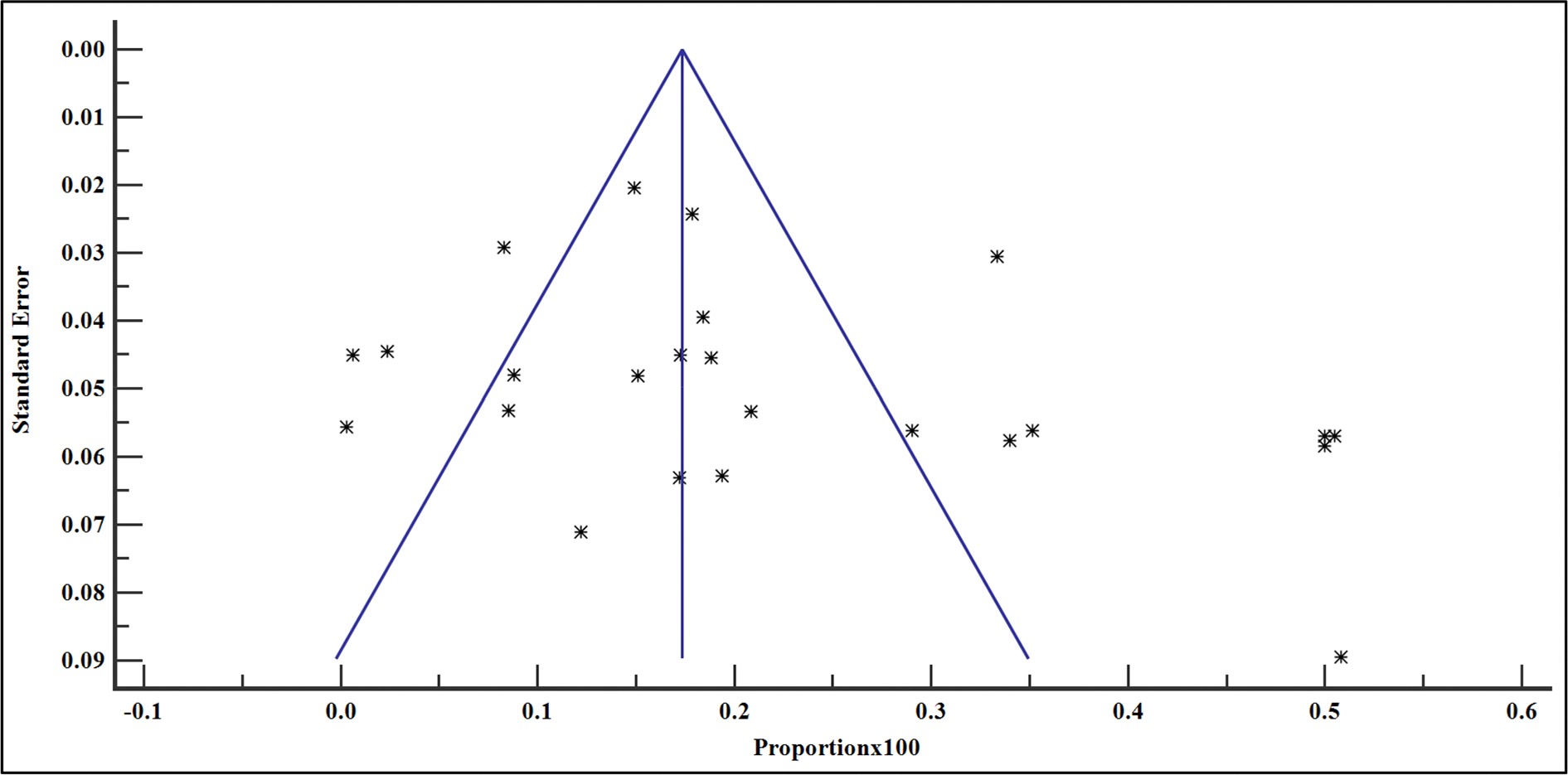
Bias assessment plot for sensitivity analysis of studies included in the data synthesis with the study which reported the largest sample size omitted.

Furthermore, there was no evidence of publication bias by both Egger’s and Begg’s tests after omitting the study by Kamau (37] (*p* > .05) (Table 2). This was confirmed by the symmetrical distribution of the data by funnel plot analysis (Figure 25).

## Discussion

The prevalence of virological failure (VF) in people with HIV is a major concern. Here, we report a pooled prevalence of 19.4%, higher than the global estimates of VF in high-income countries such as North America, China and Europe, where rates remain below 10% [40,41]. Our findings align with previous systematic reviews indicating that sub-Saharan Africa, particularly East Africa, bears a disproportionate burden of VF, with prevalence ranging between 15% and 30% [6,42]. This disparity can be attributed to multiple factors, including differences in ART regimens, healthcare infrastructure, and viral load monitoring systems. High-income countries benefit from routine viral load testing, early ART initiation and access to newer and more potent regimens [43,44], whereas resource-limited settings continue to face challenges related to treatment accessibility, adherence support and second-line therapy availability [45,46].

Pooling prevalence of VF by countries within East Africa, significant heterogeneity is observed where Tanzania’s persistently high VF rates may stem from delayed ART regimen transitions, limited viral load testing and socioeconomic barriers that impede adherence [47,48]. Notably, Kenya, Ethiopia and Uganda have demonstrated a decline in VF prevalence over time, likely due to improved treatment policies, expanded ART coverage, and stronger adherence interventions [49–51].

A temporal analysis of VF prevalence suggests an overall downward trend, with studies published between 2021 and 2023 reporting lower prevalence compared to those from 2016 to 2020. This decline coincides with the increasing use of Dolutegravir-based regimens, which have a higher genetic barrier to resistance and superior virological suppression rates compared to older non-nucleoside reverse transcriptase inhibitors (NNRTIs) [3,52,53]. The decline in virological failure can also be attributed to increased awareness and wider sensitisation that enhances adherence to drugs over the years.

Age-stratified analysis revealed that adults had the highest VF prevalence compared to children and adolescents, a finding that contrasts with previous studies suggesting that paediatric populations often experience poorer treatment outcomes [54,55]. Viral non-suppression among children has been attributed to caregiver dependency and challenges with paediatric ART formulations [47,56]. The higher VF prevalence among adults in our analysis may reflect sample size differences, as most included studies focused on adult populations. This observation aligns with the expectation that larger evidence bases may provide more accurate and representative estimates, as smaller studies are more prone to bias and outlier results. Additionally, behavioural and social factors such as economic instability, treatment fatigue, and higher mobility among adults may contribute to lower adherence and subsequent VF [57,58].

Sociodemographic characteristics also played a significant role in predicting VF. Male patients had a 1.2-fold higher risk of VF than females, consistent with previous reports that attribute this trend to poorer healthcare-seeking behaviours, lower retention in care, and higher substance use rates among men [41,59,60]. Unmarried individuals had a 1.26-fold higher risk of VF compared to married counterparts, likely due to reduced psychosocial support, stigma-related treatment interruptions, and lower motivation for adherence. Non-formal employment was also associated with a higher risk of VF, consistent with findings where these populations face unique adherence challenges, including socioeconomic pressures [61]. Additionally, financial constraints like lack of money for transportation among those with non-formal employment often can result in delayed or missed appointments to the clinic that disrupt treatment continuity [51,62].

Among clinical factors, poor baseline health status at ART initiation was strongly associated with VF. Patients who were ambulatory at ART initiation had double the risk of VF compared to those who were fully active, suggesting that initiating ART at a more advanced disease stage negatively impacts long-term treatment success [63]. A low CD4 T-cell count at enrolment also doubled the risk of VF, reinforcing the importance of early ART initiation to prevent immune deterioration and reduce the likelihood of treatment failure [8,64]. Moreover, anaemia at ART initiation increased the risk of VF by approximately 2.5 times, highlighting the critical role of nutritional and haematologic factors in ART outcomes [65,66].

Among treatment-related factors, poor adherence emerged as the strongest predictor of VF, increasing the risk by more than twofold. This finding is consistent with multiple systematic reviews and meta-analyses across different regions, which highlight the pivotal role of adherence in achieving viral suppression [23,67,68]. However, other treatment-related factors, such as ART regimen type (first line vs. second line) and regimen changes, were not significantly associated with VF, suggesting that adherence plays a more critical role in treatment outcomes than regimen type alone.

Meta-regression analysis revealed promising trends, showing an overall decline in VF prevalence over time, particularly in Ethiopia and Uganda, where expanded ART programs and improved adherence interventions have led to better virological outcomes [49,69]. Conversely, Tanzania reported an increasing trend in VF prevalence, highlighting the urgent need for targeted interventions to improve adherence and optimise ART regimens [47]. Furthermore, while VF prevalence has declined among adults, an increasing trend was observed among children and individuals on first-line ART, indicating that paediatric treatment programs require additional focus, particularly in terms of early identification of treatment failure and timely regimen switches [56,70].

## Conclusion

The findings from this systematic review and meta-analysis highlight the persistent challenge of VF in East Africa, despite notable improvements in ART accessibility and adherence interventions. While the overall prevalence of VF appears to be declining, significant disparities remain across countries, age groups and risk factors. Even a VF rate of nearly 20% is concerning, emphasising the importance of a multifaceted approach including improved viral load monitoring, enhanced ART adherence and targeted interventions that address context-specific drivers of treatment failure, such as geographic location, gender, age group, marital status and literacy levels. These efforts are essential steps towards achieving the UNAIDS 95–95–95 targets where 95% of people living with HIV know their status, 95% of diagnosed individuals access treatment, and 95% of those on treatment achieve viral suppression, ultimately contributing to the goal of ending AIDS by 2030 [3,71,72].

### Limitation of study

This study did not control for cofounders as this was beyond the scope of our meta-analysis, rather we compared subgroups to establish the relative risk of virological failure.

## Supplementary Material

yhct_a_2490774_sm6616

yhct_a_2490774_sm6617

Supplemental data for this article can be accessed online at https://doi.org/10.1080/25787489.2025.2490774.

## Figures and Tables

**Table 1.** General characteristics of the eligible studies for inclusion in the data synthesis.

First author [Ref.]	Country	Study group	Study design	Sample size	Prevalence	Q s

Nsanzimana et al. [18]	Rwanda	Adult on SLART	Retrospective cohort	1688	301	7
Hawkins et al. [19]	Tanzania,	ART-naive, adults	Cohort	2403	358	4
Tadesse et al. [10]	Ethiopia	Children on FLART	Prospective cohort	484	91	6
Bayleyegn et al. [20]	Ethiopia	Children receiving Art	Cross-sectional	253	49	6
Abera et al. [21]	Ethiopia	Children and adolescents on FLART	Prospective cohort	492	85	5
Mulisa et al. [22]	Ethiopia	Adult FLART	Cross-sectional	350	73	6
Huibers et al. [23]	Uganda	Children on FLART	Prospective cohort	316	111	6
Mamo et al. [24]	Ethiopia	Adult and adolescent on ART	Retrospective cohort	641	118	4
Mziray et al. [25]	Tanzania	Individuals failing FLART	Case-control	124	63	6
Agegnehu et al. [26]	Ethiopia	Adults on FLART	Retrospective cohort	490	3	6
Kityo et al. [27]	Uganda	Children initiating on FLART	Prospective cohort	317	92	7
Gunda et al. [9]	Tanzania	Adult on SLART	Case-control	197	24	7
Andarge et al. [28]	Ethiopia	Adult on FLART	Retrospective cohort	323	1	6
Genet et al. [12]	Ethiopia	People FLART	Cross-sectional	430	65	7
Ahmed et al. [29]	Ethiopia	Adult on FLART	Case-control	308	154	6
Mengistu et al. [30]	Eritrea	Adults on FLART	Case-control	1068	356	8
Omooja et al. [31]	Uganda	Adults on FLART	Observational	1169	97	5
Bitwale et al. [32]	Tanzania	Children and adolescents on ART	Cross-sectional	300	102	5
Milne et al. [33]	Kenya	Participants on FLART	RCT	352	30	5
Namale et al. [34]	Uganda	Female sex workers on ART	Cross-sectional	432	38	6
Bogale et al. [35]	Ethiopia	Patients on FLART	Case-control study	292	146	6
Misasew et al. [36]	Ethiopia	Children on FLART	Retrospective cohort	250	43	5
Kamau [37]	Kenya	People on ART	Cross-sectional	16,340	2156	6
Nanfuka et al. [38]	Uganda	Participants on FLART	Prospective cohort	503	12	5
Plymoth et al. [39]	Ethiopia	Adults and adolescents on ART	Case-control study	307	155	7

*Note:* SLART: second-line antiretroviral treatment, FLART: first-line antiretroviral treatment, PLLV: persistent low-level viremia, DRO Democratic Republic of congo, RCT: Randomised controlled trial.

**Table 2.** Overall, single study effect and subgroup analysis of the pooled prevalence of VF from 2016 to 2023 In East Africa.

Group	Category	No	Sample size	Analysis of the prevalence of virological failure		Analysis of heterogeneity			Analysis of the publication bias	
Variable				Prevalence VF % (95%CI)	*p* Value	*I*^2^ %	P het	Model	Egger’s test	Begg’s test

Overall		25	29,829	19.40 (15.20–24.00) Ref		98.51	<0.001	Random	0.118	0.076
One study effect		24	13,489	19.80 (14.50–25.60)	.331	98.48	<0.001	Random	0.313	0.059
Country	Tanzania	4	3024	26.50 (13.20–42.40) Ref		97.61	<0.001	Random	0.274	0.174
	Ethiopia	12	4620	20.20 (10.90–31.42)	<.001	98.74	<0.001	Random	0.270	0.217
	Kenya	2	16,692	11.11 (7.03–15.97)	<.001	86.59	0.006	Random	0.001	0.317
	Uganda	5	2737	14.51 (5.37–27.10)	<.001	98.44	<0.001	Random	0.297	0.050
Age-groups	Adults	9	21,118	32.70 (23.10–43.10) Ref		99.09	<0.001	Random	0.001	0.210
	Children	5	1794	14.99 (4.70–29.70)	<.001	98.29	<0.001	Random	0.346	1.000
	Adults and adolescents	2	826	1.29 (0.06–4.08)	<.001	84.69	0.011	Random	0.001	0.317
	Children and adolescent	2	689	15.20 (10.62–20.30)	<.001	63.98	0.096	Random	0.001	0.317
	General population	6	2999	16.96 (8.96–26.90)	<.001	97.61	0.001	Random	0.136	0.039
Year of publication	2016–2020	12	8043	24.50 (15.80–34.30) Ref		98.87	<0.001	Random	0.118	0.039
	2021–2023	13	21,786	15.28 (10.80–20.50)	<.001	97.97	<0.001	Random	0.592	0.901

**Table 3:** summary meta-analysis of the socio-demographic characteristics associated with VF among the people with HIV on first-line antiretroviral treatment.

Sub-group	Category	No	Sample size	Analysis of the prevalence of virological failure		Analysis of heterogeneity			Analysis of the publication bias	
Variable				Prevalence VF % (95% CI)	*p* Value	*I*^2^%	*P* het	Model	Egger’s test	Beggs test

Sex	Male	25	9316	30.88 (25.20–36.81) Ref		96.18	<0.001	Random	0.001	0.045
	Female	25	16,859	26.23 (21.07–31.70)	<.001	97.07	<0.001	Random	0.002	0.045
Marital status	Married	14	3859	19.32 (13.60–25.69) Ref		95.28	<0.001	Random	0.370	0.250
	Not married	14	3547	28.24 (19.45–37.96)	<.001 -	97.17	<0.001	Random	0.300	0.547
Education	Primary and below	11	3268	32.96 (19.22–48.39) Ref		98.67	<0.001	Random	0.070	0.243
	Secondary and above	11	1735	25.67 (16.75–35.75)	<.001	94.79	<0.001	Random	0.790	0.209
Employment	Formal employment	8	1362	22.59 (14.67–31.66) Ref		91.98	<0.001	Random	0.270	0.621
	Non formal employment	8	1976	47.16 (28.90–65.83)	<.001	98.53	<0.001	Random	0.120	0.216
Status disclosure	Disclosed	5	1150	20.60 (9.88–34.07) Ref		95.91	<0.001	Random	0.940	1.000
	Not disclosed	5	629	32.55 (7.18–65.50)	<.001 -	98.58	<0.001	Random	0.210	0.624
Location	Urban	4	772	51.16 (8.90–92.33) Ref		99.41	<0.001	Random	0.380	0.497
	Rural	4	733	18.86 (9.76–30.12)	<.001	92.17	<0.001	Random	0.570	0.174
HIV status of parent/caregiver	Positive	3	506	32.79 (10.78–59.86) Ref		97.40	<0.001	Random	0.060	0.117
	Negative	3	201	23.19 (8.47–42.43)	.012	88.08	<0.001	Random	0.680	0.602

**Table 4.** Summary meta-analysis of the clinical-related characteristics associated with VF among the paediatrics and adults with HIV.

Sub-group	Category	No	Analysis of the prevalence of virological failure			Analysis of heterogeneity			Analysis of the publication bias	
Variable			Sample size	Prevalence VF % (95%CI)	*p* Value	*I*^2^%	*P* het	Model	Eggers test	Beggs test

Functional status	Working/fully active	5	1602	20.10 (9.16–33.80) Ref		97.46	<0.001	Random	0.269	0.327
	Ambulatory	5	342	44.70 (29.30–60.50)	<.001	85.30	<0.001	Random	0.680	1.000
BMI	Underweight	4	556	34.90 (22.50–48.60) Ref		90.06	<0.001	Random	0.741	1.000
	Normal and above	4	2782	31.50 (19.50–44.90)	.115	97.74	<0.001	Random	0.213	0.174
CD4 T-cell count	Low	13	2975	35.10 (25.10–45.80) Ref		96.81	<0.001	Random	0.318	0.222
	Normal	13	2990	18.30 (11.20–26.60)	<.001	96.59	<0.001	Random	0.326	0.464
Anaemia	Anaemic	5	402	52.20 (37.90–66.30) Ref		87.59	<0.001	Random	0.828	0.624
	Not anaemic	5	1287	23.60 (11.80–38.10)	<.001	96.88	<0.001	Random	0.291	0.624
WHO staging	Stage I/II	11	3324	20.30 (13.20–28.50) Ref		96.23	<0.001	Random	0.957	0.586
	Stage III/IV	11	1806	44.20 (30.40–58.40)	<.001	97.07	<0.001	Random	0.026	0.139
TB	HIV-TB co-infection	6	557	24.30 (17.0–32.60) Ref		78.28	<0.001	Random	0.511	0.573
	HIV mono-infection	6	3010	17.90 (9.40–28.40)	<.001	97.59	<0.001	Random	0.865	0.851
Other opportunistic infections	Positive	6	792	20.50 (12.70–29.50) Ref		88.68	<0.001	Random	0.447	0.243
	Negative	6	1461	16.02 (8.70–25.00)	.008	94.42	<0.001	Random	0.248	0.189

**Table 5.** summary of the treatment-related characteristics associated with prevalence of VF among the people with HIV on first-line antiretroviral treatment.

Sub-group	Category	No	Analysis of the prevalence of virological failure			Analysis of heterogeneity			Analysis of the publication bias	
Variable			Sample size	Prevalence VF %(95%CI)	*p* Value	*I*^2^%	*p* het	Model	Egger’s test	Beggs test

First-line regimen	EFV-based	9	1835	23.70 (12.60–37.10) (Ref)		96.98	<0.001	Random	0.058	0.144
	NVP-based	9	1471	27.70 (17.80–38.70)	.009	94.94	<0.001	Random	0.152	0.211
Regimen change	Yes	6	1572	25.80 (10.40–45.30) (Ref)		98.21	<0.001	Random	0.075	0.573
	No	6	1929	26.00 (14.40–39.70)	.893	97.31	<0.001	Random	0.540	0.851
Second-line regimen	LPV-based	2	1248	15.99 (14.00–18.20) (Ref)		0.00	0.520	Fixed	0.001	0.317
	ATV-based	2	638	14.13 (3.50–30.20)	.289	86.14	0.007	Random	0.001	0.317
Adherence	Poor	19	2077	41.76 (32.90–50.90) (Ref)		93.81	<0.001	Random	0.260	0.278
	Good	19	4483	23.40 (16.50–31.18)	<.001	96.98	<0.001	Random		0.064

**Table 6.** Relative risk meta-analysis of the socio-demographic factors associated with VF among people with HIV on ART.

Sub-group	No	Analysis of the relative risk		Analysis of heterogeneity			Analysis of the publication bias	
Variable		Relative risk (95%CI)	*p* Value	*I*^2^% (95%CI)	*p* het	Model	Egger’s test	Beggs test

Male sex	25	1.20 (1.08–1.34)	.001*	65.48 (47.40–77.40)	<0.001	Random	0.189	0.161
Unmarried	14	1.26 (1.09–1.45)	.002*	52.98 (13.71–74.38)	0.010	Random	0.723	0.171
Non-formal education	11	1.12 (0.78–1.61)	.528	90.71 (85.40–94.08)	<0.001	Random	0.892	0.938
Non-formal employment	8	1.9 (1.18–3.06)	.008*	92.73 (1.18–3.06)	0.001	Random	0.242	0.621
No HIV status disclosure	5	1.32 (0.67–2.59)	.423	90.91 (81.74–95.48)	<0.001	Random	0.978	0.624
Rural location	4	2.06 (0.46–9.23)	.345	97.91 (96.51–98.75)	<0.001	Random	0.322	0.174
Positive HIV status of caregiver	3	1.38 (0.65–2.91)	.399	81.33 (41.90–94.00)	0.005	Random	0.784	0.602

Asterisk (*) denotes *p*-values (≤ .05) that are statistically significant.

**Table 7.** Relative risk meta-analysis of the clinical factors associated with VF among people with HIV on ART.

Sub-group	No	Analysis of the relative risk		Analysis of heterogeneity			Analysis of the publication bias	
Variable		Relative risk (95%CI)	*p* Value	*I*^2^% (95%CI)	*p* het	Model	Egger’s test	Beggs test

Functional status	4	2.15 (1.30–3.55)	.003*	88.02 (74.58–94.3)	<0.001	Random	0.161	0.624
BMI	4	1.11 (0.98–1.27)	.115	0.00 (0.00–81.76)	0.547	Fixed	0.988	0.497
CD4 count	13	2.03 (1.39–2.97)	<.001*	92.02 (88.16–94.63)	<0.001	Random	0.460	0.714
Anaemia	5	2.36 (1.41–3.93)	.001*	92.17 (84.71–95.99)	<0.001	Random	0.191	0.327
WHO staging	11	0.92 (0.20–4.29)	.913	99.61 (99.54–99.66)	<0.001	Random	0.095	0.586
HIV/TB co-infection	6	1.43 (0.94–2.16)	.094	81.78 (61.16–91.45)	<0.001	Random	0.284	0.348
Infection with other Ois	6	1.36 (0.67–2.76)	.397	92.24 (85.86 to 95.74)	<0.001	Random	0.918	0.851

Asterisk (*) denotes *p*-values (≤ .05) that are statistically significant.

**Table 8.** Relative risk meta-analysis of the treatment-related factors associated with VF among people with HIV on ART.

Sub-group	No	Analysis of the relative risk		Analysis of heterogeneity			Analysis of the publication bias	
Variable		Relative risk (95%CI)	*p* Value	*I*^2^% (95%CI)	*p* het	Model	Egger’s test	Beggs test

Regimen (NVP vs EFV)	9	1.22 (0.80–1.87)	.349	86.28 (75.93–92.18)	<0.001	Random	0.623	0.677
Changed regimen	6	0.92 (0.37–2.30)	.852	97.31 (95.8–98.3)	<0.001	Random	0.778	0.573
Regimen (LPV vs LTV)	2	1.11 (0.42–2.92)	.833	66.35 (0.00–92.37)	0.085	Random	0.001	0.317
Adherence	19	2.01 (1.42–2.84)	<.001	94.14 (92.14–95.64)	<0.001	Random	0.741	0.807

## References

[1] Mojola SA, Angotti N, Denardo D, The end of AIDS? HIV and the new landscape of illness in rural South Africa. Glob Public Health. 2022;17(1):13–25. 33290168 10.1080/17441692.2020.1851743 PMC8184878

[2] WHO. WHO guidelines approved by the guidelines review committee. Consolidated guidelines on the use of antiretroviral drugs for treating and preventing HIV infection: recommendations for a public health approach. World Health Organization; 2016. https://www.ncbi.nlm.nih.gov/books/NBK374318/27466667

[3] Heath K, Levi J, Hill A. The joint United Nations programme on HIV/AIDS 95–95–95 targets: worldwide clinical and cost benefits of generic manufacture. AIDS. 2021;35(Suppl 2):S197–S203. 34115649 10.1097/QAD.0000000000002983

[4] Ayele TA, Worku A, Kebede Y, Choice of initial antiretroviral drugs and treatment outcomes among HIV-infected patients in Sub-Saharan Africa: systematic review and meta-analysis of observational studies. Syst Rev. 2017;6(1):173. 28841912 10.1186/s13643-017-0567-7 PMC5574138

[5] Foka FET, Mufhandu HT. Current ARTs, virologic failure, and implications for AIDS management: a systematic review. Viruses. 2023;15(8):1732–1760. 37632074 10.3390/v15081732 PMC10458198

[6] Gupta RK, Gregson J, Parkin N, HIV-1 drug resistance before initiation or re-initiation of first-line antiretroviral therapy in low-income and middle-income countries: a systematic review and meta-regression analysis. Lancet Infect Dis. 2018;18(3):346–355. 29198909 10.1016/S1473-3099(17)30702-8 PMC5835664

[7] Namaganda MM, Sendagire H, Kateete DP, Next-generation sequencing (NGS) reveals low-abundance HIV-1 drug resistance mutations among patients experiencing virological failure at the time of therapy switching in Uganda. F1000Res. 2022;11:901.

[8] Bossard C, Schramm B, Wanjala S, High prevalence of NRTI and NNRTI drug resistance among ART-experienced, hospitalized inpatients. J Acquir Immune Defic Syndr. 2021;87(3):883–888. 33852504 10.1097/QAI.0000000000002689 PMC8191469

[9] Gunda DW, Kilonzo SB, Mtaki T, Magnitude and correlates of virological failure among adult HIV patients receiving PI based second line ART regimens in North Western Tanzania; a case control study. BMC Infect Dis. 2019;19(1):235. 30845924 10.1186/s12879-019-3852-3 PMC6407235

[10] Tadesse BT, Foster BA, Latour E, Predictors of virologic failure among a cohort of HIV-infected children in Southern Ethiopia. Pediatr Infect Dis J. 2021;40(1):60–65. 32925538 10.1097/INF.0000000000002898

[11] World Health Organization. Consolidated guidelines on the use of antiretroviral drugs for treating and preventing HIV infection: recommendations for a public health approach; 2016 [cited October 2023]. Available from: https://www.who.int/publications/i/item/9789241549684 27466667

[12] Genet A, Mekonnen Z, Yizengaw E, First line antiretroviral treatment failure and associated factors among people living with HIV in northwest Ethiopia. Afr Health Sci. 2021;21(1):263–272. 34394306 10.4314/ahs.v21i1.34 PMC8356610

[13] Wells GA, Wells G, Shea B, The Newcastle-Ottawa scale (NOS) for assessing the quality of nonrandomised studies in meta-analyses. 2014. https://www.ohri.ca/programs/clinical_epidemiology/nosgen.pdf

[14] Begg CB, Mazumdar M. Operating characteristics of a rank correlation test for publication bias. Biometrics. 1994;50(4):1088–1101. 7786990

[15] Egger M, Davey Smith G, Schneider M, Bias in meta-analysis detected by a simple, graphical test. BMJ. 1997;315(7109):629–634. 9310563 10.1136/bmj.315.7109.629 PMC2127453

[16] Gjerdevik M, Heuch I. Improving the error rates of the Begg and Mazumdar test for publication bias in fixed effects meta-analysis. BMC Med Res Methodol. 2014;14(1):109. 25245217 10.1186/1471-2288-14-109 PMC4193136

[17] Cheung MWL. Fixed- and random-effects meta-analytic structural equation modeling: examples and analyses in R. Behav Res Methods. 2014;46(1):29–40. 23807765 10.3758/s13428-013-0361-y

[18] Nsanzimana S, Semakula M, Ndahindwa V, Retention in care and virological failure among adult HIV+ patients on second-line ART in Rwanda: a national representative study. BMC Infect Dis. 2019;19(1):312. 30953449 10.1186/s12879-019-3934-2 PMC6451213

[19] Hawkins C, Ulenga N, Liu E, HIV virological failure and drug resistance in a cohort of Tanzanian HIV-infected adults. J Antimicrob Chemother. 2016;71(7):1966–1974. 27076106 10.1093/jac/dkw051 PMC4896406

[20] Bayleyegn B, Kifle ZD, Geremew D. Virological failure and associated factors among children receiving anti-retroviral therapy, northwest Ethiopia. PLoS One. 2021;16(9):e0257204. 34506553 10.1371/journal.pone.0257204 PMC8432779

[21] Abera NM, Alemu TG, Agegnehu CD. Incidence and predictors of virological failure among HIV infected children and adolescents on first-line antiretroviral therapy in east shewa hospitals, Oromia region, Ethiopia: a retrospective follow up study. PLoS One. 2023;18(11):e0289095. 38033131 10.1371/journal.pone.0289095 PMC10688895

[22] Mulisa D, Tolossa T, Bayisa L, First-line virologic-based ART treatment failure and associated factors among adult HIV positives in Southwest Shoa, Central Ethiopia. J Int Assoc Provid AIDS Care. 2022;21:23259582221111080. 35844136 10.1177/23259582221111080 PMC9297459

[23] Huibers MHW, Kityo C, Boerma RS, Long-term virological outcomes, failure and acquired resistance in a large cohort of Ugandan children. J Antimicrob Chemother. 2019;74(10):3035–3043. 31289811 10.1093/jac/dkz266

[24] Mamo A, Assefa T, Negash W, Virological and immunological antiretroviral treatment failure and predictors among HIV positive adult and adolescent clients in southeast Ethiopia. HIV AIDS. 2022;14:73–85. 10.2147/HIV.S354716 PMC8892571 35250314

[25] Mziray SR, Kumburu HH, Assey HB, Patterns of acquired HIV-1 drug resistance mutations and predictors of virological failure in Moshi, Northern Tanzania. PLoS One. 2020;15(9):e0232649. 32986709 10.1371/journal.pone.0232649 PMC7521739

[26] Agegnehu CD, Merid MW, Yenit MK. Incidence and predictors of virological failure among adult HIV patients on first-line antiretroviral therapy in Amhara regional referral hospitals; Ethiopia: a retrospective follow-up study. BMC Infect Dis. 2020;20(1):460. 32611405 10.1186/s12879-020-05177-2 PMC7329399

[27] Kityo C, Boerma RS, Sigaloff KCE, Pretreatment HIV drug resistance results in virological failure and accumulation of additional resistance mutations in Ugandan children. J Antimicrob Chemother. 2017;72(9):2587–2595. 28673027 10.1093/jac/dkx188 PMC5890670

[28] Andarge DE, Hailu HE, Menna T. Incidence, survival time and associated factors of virological failure among adult HIV/AIDS patients on first line antiretroviral therapy in St. Paul’s hospital millennium medical college-a retrospective cohort study. PLoS one. 2022;17(10):e0275204. 36227886 10.1371/journal.pone.0275204 PMC9560068

[29] Ahmed M, Merga H, Jarso H. Predictors of virological treatment failure among adult HIV patients on first-line antiretroviral therapy in Woldia and Dessie hospitals, northeast Ethiopia: a case-control study. BMC Infect Dis. 2019;19(1):305. 30943903 10.1186/s12879-019-3924-4 PMC6448227

[30] Mengistu ST, Ghebremeskel GG, Ghebrat HB, Determinants of therapy failure among adults on first-line antiretroviral therapy in Asmara, Eritrea: a multicenter retrospective matched case-control study. BMC Infect Dis. 2022;22(1):834. 36357837 10.1186/s12879-022-07797-2 PMC9650854

[31] Omooja J, Nannyonjo M, Sanyu G, Rates of HIV-1 virological suppression and patterns of acquired drug resistance among fisherfolk on first-line antiretroviral therapy in Uganda. J Antimicrob Chemother. 2019;74(10):3021–3029. 31257432 10.1093/jac/dkz261 PMC6753497

[32] Bitwale NZ, Mnzava DP, Kimaro FD, Prevalence and factors associated with virological treatment failure among children and adolescents on antiretroviral therapy attending HIV/AIDS care and treatment clinics in Dodoma municipality, Central Tanzania. J Pediatric Infect Dis Soc. 2021;10(2):131–140. 32463083 10.1093/jpids/piaa030

[33] Milne RS, Beck IA, Levine M, Low-frequency pre-treatment HIV drug resistance: effects on 2-year outcome of first-line efavirenz-based antiretroviral therapy. AIDS. 2022;36(14):1949–1958. 36305180 10.1097/QAD.0000000000003361 PMC9623471

[34] Namale G, Kamacooko O, Bagiire D, Sustained virological response and drug resistance among female sex workers living with HIV on antiretroviral therapy in Kampala, Uganda: a cross-sectional study. Sex Transm Infect. 2019;95(6):405–411. 31266818 10.1136/sextrans-2018-053854 PMC6824617

[35] Bogale B, Asefa A, Destaw A, Determinants of virological failure among patients on first line highly active antiretroviral therapy (HAART) in Southwest Ethiopia: a case-control study. Front Public Health. 2022;10:916454. 36408009 10.3389/fpubh.2022.916454 PMC9667891

[36] Misasew M, Menna T, Berhan E, Incidence and predictors of antiretroviral treatment failure among children in public health facilities of Kolfe Keranyo Sub-City, Addis Ababa, Ethiopia: institution-based retrospective cohort study. PLoS One. 2023;18(8):e0266580. 37594924 10.1371/journal.pone.0266580 PMC10437829

[37] Kamau. 2023. Prevalence and predictors of virologic failure among HIV patients on antiretroviral therapy in Makueni County: a crosssectional study. F1000Research Available from: https://f1000research.com/articles/12-879

[38] Nanfuka M, Forrest JI, Zhang W, Durability of non-nucleotide reverse transcriptase inhibitor-based first-line ART regimens after 7 years of treatment in rural Uganda: a prospective cohort study. Medicine. 2021;100(19):e25763. 34106606 10.1097/MD.0000000000025763 PMC8133171

[39] Plymoth M, Sanders EJ, Van Der Elst EM, Socio-economic condition and lack of virological suppression among adults and adolescents receiving antiretroviral therapy in Ethiopia. PLoS One. 2020;15(12):e0244066. 33320900 10.1371/journal.pone.0244066 PMC7737988

[40] Waters L, Winston A, Reeves I, BHIVA guidelines on antiretroviral treatment for adults living with HIV-1 2022. HIV Med. 2022;23(S5):3–115. 10.1111/hiv.13446 36504313

[41] Yuan D, Liu M, Jia P, Prevalence and determinants of virological failure, genetic diversity and drug resistance among people living with HIV in a minority area in China: a population-based study. BMC Infect Dis. 2020;20(1):443. 32576136 10.1186/s12879-020-05124-1 PMC7310496

[42] Agegnehu CD, Techane MA, Mersha AT, Burden and associated factors of virological failure among people living with HIV in Sub-Saharan Africa: a systematic review and meta-analysis. AIDS Behav. 2022;26(10):3327–3336. 35416596 10.1007/s10461-022-03610-y

[43] Aldous JL, Haubrich RH. Defining treatment failure in resource-rich settings. Curr Opin HIV AIDS. 2009;4(6):459–466. 20048711 10.1097/COH.0b013e328331dea5 PMC2946177

[44] Bertagnolio S, Hermans L, Jordan MR, Clinical impact of pretreatment human immunodeficiency virus drug resistance in people initiating nonnucleoside reverse transcriptase inhibitor–containing antiretroviral therapy: a systematic review and meta-analysis. J Infect Dis. 2021;224(3):377–388. 33202025 10.1093/infdis/jiaa683 PMC8328216

[45] Kippen A, Nzimande L, Gareta D, The viral load monitoring Cascade in HIV treatment programmes in Sub-Saharan Africa: a systematic review. BMC Public Health. 2024;24(1):2603. 39334013 10.1186/s12889-024-20013-x PMC11428611

[46] Lailulo Y, Kitenge M, Jaffer S, Factors associated with antiretroviral treatment failure among people living with HIV on antiretroviral therapy in resource-poor settings: a systematic review and metaanalysis. Syst Rev. 2020;9(1):292. 33308294 10.1186/s13643-020-01524-1 PMC7733304

[47] Muri L, Gamell A, Ntamatungiro AJ, Development of HIV drug resistance and therapeutic failure in children and adolescents in rural Tanzania: an emerging public health concern. AIDS. 2017;31(1):61–70. 27677163 10.1097/QAD.0000000000001273 PMC5131685

[48] Mwavika ET, Kunambi PP, Masasi SJ, Prevalence, rate, and predictors of virologic failure among adult HIV-infected clients on second-line antiretroviral therapy (ART) in Tanzania (2018–2020): a retrospective cohort study. Bull Natl Res Cent. 2024;48(1):96.

[49] Aytenew TM, Asferie WN, Ejigu N, Virological failure and associated factors among patients receiving anti-retroviral therapy in Ethiopia: a systematic review and meta-analysis. BMJ Open. 2024;14(11):e087569. 10.1136/bmjopen-2024-087569 PMC11605831 39613423

[50] Ochieng W, Kitawi RC, Nzomo TJ, Implementation and operational research: correlates of adherence and treatment failure among Kenyan patients on long-term highly active antiretroviral therapy. J Acquir Immune Defic Syndr. 2015;69(2):e49–e56. 26009836 10.1097/QAI.0000000000000580 PMC4445604

[51] Okoboi S, Mujugira A, Nekesa N, Barriers and facilitators of adherence to long-term antiretroviral treatment in Kampala, Uganda. PLOS Glob Public Health. 2025;5(3):e0004121. 40080505 10.1371/journal.pgph.0004121 PMC11906038

[52] Rolle C-P, Berhe M, Singh T, Dolutegravir/lamivudine as a first-line regimen in a test-and-treat setting for newly diagnosed people living with HIV. AIDS. 2021;35(12):1957–1965. 34115650 10.1097/QAD.0000000000002979 PMC8462441

[53] Venter WD, Sokhela S, Simmons B, Dolutegravir with emtricitabine and tenofovir alafenamide or tenofovir disoproxil fumarate versus efavirenz, emtricitabine, and tenofovir disoproxil fumarate for initial treatment of HIV-1 infection (ADVANCE): week 96 results from a randomised, phase 3, non-inferiority trial. Lancet HIV. 2020;7(10):e666–e676. 33010240 10.1016/S2352-3018(20)30241-1

[54] Boerma RS, Boender TS, Bussink AP, Suboptimal viral suppression rates among HIV-infected children in low-and middle-income countries: a meta-analysis. Clin Infect Dis. 2016;63(12):1645–1654. 27660236 10.1093/cid/ciw645

[55] Jenabian M-A, Costiniuk CT, Mboumba Bouassa R-S, Tackling virological failure in HIV-infected children living in Africa. Expert Rev Anti Infect Ther. 2015;13(10):1213–1223. 26204960 10.1586/14787210.2015.1068117

[56] Perez F, Leroy V. Pediatric adherence to antiretroviral therapy in resource-poor settings: challenges and future perspectives. HIV Ther. 2009;3(3):213–219.

[57] Gumede SB, Wensing AMJ, Lalla-Edward ST, Predictors of treatment adherence and virological failure among people living with HIV receiving antiretroviral therapy in a South African rural community: a sub-study of the ITREMA randomised clinical trial. AIDS Behav. 2023;27(12):3863–3885. 37382825 10.1007/s10461-023-04103-2 PMC10598166

[58] Wang J, Geng L. Effects of socioeconomic status on physical and psychological health: lifestyle as a mediator. Int J Environ Res Public Health. 2019;16(2):281–290. 30669511 10.3390/ijerph16020281 PMC6352250

[59] Hlongwa M, Basera W, Hlongwana K, Linkage to HIV care and early retention in HIV care among men in the ‘universal test-and-treat’ era in a high HIV-burdened district, KwaZulu-Natal, South Africa. BMC Health Serv Res. 2024;24(1):384. 38561736 10.1186/s12913-024-10736-3 PMC10985849

[60] Taylor-Smith K, Tweya H, Harries A, Gender differences in retention and survival on antiretroviral therapy of HIV-infected adults in Malawi. Malawi Med J. 2010;22(2):49–56. 21614882 10.4314/mmj.v22i2.58794 PMC3345762

[61] Niyonsenga PM, Habimana A, Nsanzabera C. Prevalence and factors associated with non-adherence to antiretroviral therapy in HIV patients at Kibungo referral hospital, in Rwanda. 2025; medRxiv https://www.medrxiv.org/content/10.1101/2025.01.02.25319892v1.

[62] Amuri M, Mitchell S, Cockcroft A, Socio-economic status and HIV/AIDS stigma in Tanzania. AIDS Care. 2011;23(3):378–382. 21347901 10.1080/09540121.2010.507739

[63] Lilian RR, Rees K, McIntyre JA, Same-day antiretroviral therapy initiation for HIV-infected adults in South Africa: analysis of routine data. PLoS One. 2020;15(1):e0227572. 31935240 10.1371/journal.pone.0227572 PMC6959580

[64] SeyedAlinaghi S, Afsahi AM, Moradi A, Current ART, determinants for virologic failure and implications for HIV drug resistance: an umbrella review. AIDS Res Ther. 2023;20(1):74. 37884997 10.1186/s12981-023-00572-6 PMC10604802

[65] Kiragga AN, Mubiru F, Kambugu AD, A decade of antiretroviral therapy in Uganda: what are the emerging causes of death? BMC Infect Dis. 2019;19(1):77. 30665434 10.1186/s12879-019-3724-x PMC6341568

[66] Mengistu ST, Yohannes A, Issaias H, Antiretroviral therapy regimen modification rates and associated factors in a cohort of HIV/AIDS patients in Asmara, Eritrea: a 16-year retrospective analysis. Sci Rep. 2023;13(1):4183. 36918596 10.1038/s41598-023-30804-8 PMC10015006

[67] Bondarchuk CP, Mlandu N, Adams T, Predictors of low antiretroviral adherence at an urban South African clinic: a mixed-methods study. South Afr J HIV Med. 2022;23(1):1343. 35284095 10.4102/sajhivmed.v23i1.1343 PMC8905451

[68] Cresswell FV, Lamorde M. Implementation of long-acting antiretroviral therapy in low-income and middle-income countries. Curr Opin HIV AIDS. 2022;17(3):127–134. 35439787 10.1097/COH.0000000000000732

[69] Hemelaar J, Elangovan R, Yun J, Global and regional molecular epidemiology of HIV-1, 1990–2015: a systematic review, global survey, and trend analysis. Lancet Infect Dis. 2019;19(2):143–155. 30509777 10.1016/S1473-3099(18)30647-9

[70] Dubrocq G, Rakhmanina N, Phelps BR. Challenges and opportunities in the development of HIV medications in pediatric patients. Paediatr Drugs. 2017;19(2):91–98. https://link.springer.com/article/10.1007/s40272-016-0210-4 28074348 10.1007/s40272-016-0210-4

[71] Frescura L, Godfrey-Faussett P, Feizzadeh AA, Achieving the 95 95 95 targets for all: a pathway to ending AIDS. PLoS One. 2022;17(8):e0272405. 35925943 10.1371/journal.pone.0272405 PMC9352102

[72] Kumwenda M, Skovdal M, Wringe A, Exploring the evolution of policies for universal antiretroviral therapy and their implementation across three Sub-Saharan African countries: findings from the SHAPE study. Glob Public Health. 2021;16(2):227–240. 33275872 10.1080/17441692.2020.1851386 PMC7612916

