# Supplementary material for: Prevalence and predictors of virological failure among the people living with HIV on antiretroviral treatment in East Africa: evidence from a systematic review with meta-analysis and meta-regression of published studies from 2016 to 2023
Source: HIV Res Clin Pract. Author manuscript; Available in PMC 2025 Dec 1. (PMC12182973; doi:10.1080/25787489.2025.2490774)
Supplement: yhct_a_2490774_sm6616 [file NIHMS2086789-supplement-yhct_a_2490774_sm6616.docx]

| **Section and Topic** | **Item #** | **Checklist item** | **Location where item is reported** |
| --- | --- | --- | --- |
| **TITLE** | | |  |
| Title | 1 | **Prevalence and predictors of virological failure among the HIV infected persons on antiretroviral treatment in East Africa: Evidence from a systematic review with meta-analysis and meta-regression of published studies from 2016 to 2023** | Tittle page |
| **ABSTRACT** | | |  |
| Abstract | 2 | 1. The main objective(s) the systematic review and meta-analysis intended to address have been highlighted 2. The inclusion and exclusion criteria for the systematic review and meta-analysis have been specified 3. The information sources of the systematic review and meta-analysis have been specified 4. The methods used to present and synthesize results have been specified. 5. Results of the systematic review and meta-analysis have been presented 6. Discussion of results has been comprehensively done | Page 1  Page 1  Page 1  Page 1  Page 1  Page 1 |
| **INTRODUCTION** | | |  |
| Rationale | 3 | The rationale for the systematic review and meta-analysis in content of the existing knowledge on the prevalence and predictors of virological failure in the context of existing knowledge on HIV has been described. | Page 2-3 |
| Objectives | 4 | The main objective(s) of the systematic review and meta-analysis have been highlighted | Page 3 |
| **METHODS** | | |  |
| Eligibility criteria | 5 | The inclusion and exclusion criteria have been specified. | Page 5 |
| Information sources | 6 | 1. The databases sources that were searched or consulted to identify studies have been specified 2. The time range when the sources were searched has been specified. | Page 5 |
| Search strategy | 7 | The search strategies used for all databases have been specified. | Page 5 |
| Selection process | 8 | The methods used to decide whether a study meets the inclusion criteria of the review, including how many reviewers screened each record and each report that was retrieved have been specified. | Page 5 |
| Data collection process | 9 | The methods that were used to collect data from reports, including how many reviewers collected data from each report, whether they worked independently, any processes that were used for obtaining or confirming data from study investigators have been specified. | Page 5 |
| Data items | 10a | Not applicable |  |
|  | 10b | Not applicable |  |
| Study risk of bias assessment | 11 | The methods used to assess risk of bias in the included studies, including details of the tool(s) that used, how many reviewers assessed each study and whether they worked independently have been specified | Page 6 |
| Effect measures | 12 | The effect measure(s) (e.g. relative proportion) that were used in the synthesis or presentation of results for each outcome have been specified. | Page 6 |
| Synthesis methods | 13a | The processes used to decide which studies were eligible for each synthesis have been described | Page 6 |
|  | 13b | The methods used to prepare the data for presentation or synthesis have been described | Pages 6 |
|  | 13c | The methods used to tabulate or visually display results of individual studies and syntheses have been described. | Pages 6 |
|  | 13d | The methods used to synthesize results and a rationale for the choice(s), the method(s) used to identify the presence and extent of statistical heterogeneity and the software package used have been described. | Pages 6 |
|  | 13e | The methods used to explore possible causes of heterogeneity among study results (e.g. subgroup analysis, meta-regression) have been described. | Page 6 |
|  | 13f | The sensitivity analysis (sub-group analysis) conducted to assess robustness of the synthesized results has been described | Page |
| Reporting bias assessment | 14 | The methods to be used to assess risk of bias have not been described because of the would many forest plots |  |
| Certainty assessment | 15 | The methods used to assess certainty (or confidence) in the body of evidence for an outcome have been described. | Page 9-12 |
| **OTHER INFORMATION** | | |  |
| Registration and protocol | 24a | Registration number **CRD42023468918**, International Prospective Register of Systematic Reviews (PROSPERO), University of York Center for Reviews and Dissemination. | Page 4 |
|  | 24b | The protocol is available at  <https://www.crd.york.ac.uk/>PROSPERO | Page 4 |
|  |  |  |  |
| Support | 25 | Research reported in this publication was supported by the Fogarty International centre of the National Institutes of Health under award number U2RTW012116. The content is solely the responsibility of the authors and does not necessarily represent the official views of the National Institutes of Health |  |
| Competing interests | 26 | The authors of the protocol declare that they have no competing interests. |  |
| Availability of data, code and other materials | 27 | Not applicable |  |

*From:*  Page MJ, McKenzie JE, Bossuyt PM, Boutron I, Hoffmann TC, Mulrow CD, et al. The PRISMA 2020 statement: an updated guideline for reporting systematic reviews. BMJ 2021;372:n71. doi: 10.1136/bmj.n71

For more information, visit: <http://www.prisma-statement.org/>
