# Supplementary material for: Prevalence and predictors of virological failure among the people living with HIV on antiretroviral treatment in East Africa: evidence from a systematic review with meta-analysis and meta-regression of published studies from 2016 to 2023
Source: HIV Res Clin Pract. Author manuscript; Available in PMC 2025 Dec 1. (PMC12182973; doi:10.1080/25787489.2025.2490774)
Supplement: yhct_a_2490774_sm6617 [file NIHMS2086789-supplement-yhct_a_2490774_sm6617.docx]

S2 Table B. Detailed Newcastle-Ottawa Scale of each included study.

| **Study**  **(author, year)** | **Selection** | | | | **Comparability** | | **Outcome** | | | **Total quality score** |
| --- | --- | --- | --- | --- | --- | --- | --- | --- | --- | --- |
| Study | Representativeness of exposed cohort | Selection of external control | Ascertainment of exposure | Outcome of interest not present at start of study | Main factor | Additional factor | Assessment of outcomes | Sufficient follow up time | Adequacy of follow-up | (QS) |
| Nsanzimana et al. (2019) | 1 | 0 | 1 | 1 | 1 | 0 | 1 | 1 | 1 | 7 |
| Hawkins et al. (2016) | 1 | 0 | 1 | 0 | 1 | 0 | 1 | 1 | 0 | 4 |
| Tadesse et al. (2021) | 1 | 0 | 1 | 0 | 1 | 0 | 1 | 1 | 1 | 6 |
| Bayleyegn et al. (2021) | 1 | 0 | 1 | 0 | 1 | 0 | 1 | 1 | 1 | 6 |
| Abera et al. (2023) | 1 | 0 | 1 | 0 | 1 | 1 | 1 | 0 | 0 | 5 |
| Mulisa et al. (2022) | 1 | 0 | 1 | 0 | 1 | 1 | 1 | 0 | 0 | 6 |
| Huibers et al. (2019) | 0 | 0 | 1 | 0 | 1 | 1 | 1 | 1 | 1 | 6 |
| Mamo et al. (2022) | 1 | 0 | 1 | 0 | 1 | 0 | 1 | 0 | 0 | 4 |
| Mziray et al. (2020) | 0 | 1 | 1 | 0 | 1 | 1 | 0 | 1 | 1 | 6 |
| Agegnehu et al. (2020) | 1 | 1 | 0 | 0 | 1 | 0 | 1 | 1 | 1 | 6 |
| Kityo et al. (2017) | 1 | 0 | 1 | 0 | 1 | 1 | 1 | 1 | 1 | 7 |
| Gunda et al. (2019) | 1 | 0 | 1 | 0 | 1 | 1 | 1 | 1 | 1 | 7 |
| Andarge et al. (2022) | 1 | 0 | 1 | 0 | 1 | 1 | 1 | 1 | 0 | 6 |
| Genet et al. (2021) | 1 | 0 | 1 | 0 | 1 | 1 | 1 | 1 | 1 | 7 |
| Ahmed et al. (2019) | 1 | 1 | 1 | 0 | 1 | 1 | 1 | 0 | 0 | 6 |
| Mengistu et al. (2022) | 1 | 0 | 1 | 0 | 1 | 1 | 1 | 1 | 1 | 8 |
| Omooja et al. (2019) | 1 | 0 | 1 | 0 | 1 | 1 | 1 | 0 | 0 | 5 |
| Bitwale et al. (2021) | 1 | 0 | 1 | 0 | 1 | 1 | 1 | 0 | 0 | 5 |
| Milne et al. (2022) | 1 | 0 | 1 | 1 | 1 | 1 | 1 | 1 | 1 | 8 |
| Namale et al. (2019) | 0 | 0 | 1 | 1 | 1 | 1 | 1 | 1 | 0 | 6 |
| Bogale et al. (2022) | 0 | 1 | 1 | 1 | 1 | 1 | 1 | 0 | 0 | 6 |
| Misasew et al. (2023) | 0 | 0 | 1 | 0 | 1 | 1 | 1 | 0 | 0 | 5 |
| Kamau (2023), | 1 | 0 | 1 | 0 | 1 | 1 | 0 | 1 | 1 | 6 |
| Nanfuka et al. (2021) | 0 | 1 | 0 | 0 | 1 | 1 | 0 | 1 | 1 | 5 |
| Plymoth et al. (2020) | 1 | 0 | 1 | 1 | 1 | 0 | 1 | 1 | 1 | 7 |
